# Unlocking Extra Flexibility of Soft Porous Crystals via Hydrogen Bonding Interaction for Multistep Acetylene Sorption

**DOI:** 10.1002/smll.74777

**Published:** 2026-07-25

**Authors:** Christophe Lavenn, Nobuhiko Hosono, Ken‐ichi Otake, Susan Sen, Shinpei Kusaka, Tomoe Watanabe, Hirotaka Ashitani, Shogo Kawaguchi, Hiroki Ishibashi, Yoshiki Kubota, Ryotaro Matsuda, Susumu Kitagawa

**Affiliations:** ^1^ Air Liquide Innovation Campus Paris Les Loges‐en‐Josas France; ^2^ Department of Applied Chemistry Graduate School of Engineering The University of Tokyo Tokyo Japan; ^3^ Institute for Integrated Cell‐Material Sciences Kyoto University Institute for Advanced Study Kyoto University Kyoto Japan; ^4^ Department of Materials Chemistry Graduate School of Engineering Nagoya University Nagoya Japan; ^5^ Department of Physics Graduate School of Science Osaka Metropolitan University Osaka Japan; ^6^ Japan Synchrotron Radiation Research Institute (JASRI) Hyogo Japan

**Keywords:** acetylene, adsorption, hydrogen bonding, metal‐organic frameworks, porous coordination polymers

## Abstract

Flexible porous coordination polymers (PCPs) have garnered significant attention as promising materials for efficient gas storage due to their ability to exhibit large adsorption changes within narrow pressure ranges, although the chemical design principles necessary to control such behavior remain largely undeveloped. In this study, we demonstrate that minor chemical modifications to the ligand constituting PCPs can substantially change their flexibility and gas storage properties. By substituting the conventional isophthalate (ipa) ligand with pyridine‐3,5‐dicarboxylate (pyda) in a typical CID (coordination polymer with interdigitated framework)‐type flexible PCPs, we achieved a significant enhancement in acetylene storage capacity and structural flexibility. The nitrogen atom on the pyda ligand acts as a hydrogen bond acceptor, facilitating strong interactions with acetylene molecules and activating latent structural flexibility. This modification enabled 276% pore volume expansion and a fivefold increase in acetylene adsorption compared to the ipa‐based PCP, while also inducing unique multistep gate‐opening behavior. Furthermore, the framework demonstrated selective adsorption for acetylene over ethylene and ethane, despite the minimal structural difference in the ligands. These findings highlight the critical role of hydrogen bonding in stabilizing and unlocking hidden framework phases, providing valuable insights into ligand design strategies for improving gas storage performance in flexible PCPs.

## Graphical abstract

Minor ligand modification in a CID‐type flexible PCP induces a fivefold increase in acetylene uptake and > 270% pore expansion. By replacing isophthalate with pyridine‐3,5‐dicarboxylate, the framework exhibits hydrogen‐bond‐assisted multistep gate‐opening behavior and selective adsorption of acetylene over other C2 hydrocarbons. These findings offer new insights into molecular‐level design strategies for enhancing flexible PCP performance.

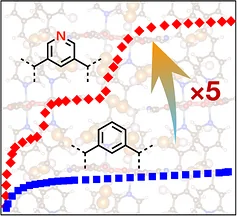

## 1 Introduction

Porous coordination polymers (PCPs), also known as metal–organic frameworks (MOFs), have attracted considerable attention because their framework structures and host–guest interactions can be systematically tuned at the molecular level [1, 2, 3, 4, 5]. Among them, flexible PCPs are particularly intriguing because they can undergo guest‐induced structural transformations, often exhibiting gate‐opening adsorption behavior that enables large adsorption changes within narrow pressure ranges [6, 7]. This adsorption profile is characterized by abrupt adsorption and desorption at specific pressures known as gate‐opening (GO) and gate‐closing (GC) pressures, respectively. Such unique adsorption responses have been widely explored for gas storage, separation, sensing, and molecular recognition applications.

Despite extensive studies on flexible PCPs, rational design strategies that enable precise control over framework flexibility and adsorption‐induced phase transitions remain underdeveloped. In particular, it is still challenging to predict how subtle modifications of ligand structures influence the balance between the deformation energy required for framework transformation and the stabilization energy provided by guest adsorption, which ultimately governs gate‐opening behavior and determines the accessible pore volume in the adsorbed state [7].

Acetylene is an attractive probe molecule for investigating these phenomena because its strong adsorption affinity and directional intermolecular interactions can induce pronounced structural responses in flexible frameworks. Several PCPs have been reported to stabilize acetylene molecules by isolating them within confined pores and by reducing the electron density of the triple bond through coordination and hydrogen‐bonding interactions [8, 9, 10, 11, 12]. More recently, the modulation of hydrogen‐bonding interactions within the nanopores of PCPs has emerged as a new strategy for controlling gas diffusion, thereby facilitating effective separation of acetylene gases [13, 14, 15]. These observations suggest that hydrogen‐bonding motifs may provide a powerful handle for controlling adsorption‐induced structural transformations in flexible PCPs, although their role in governing framework flexibility remains largely unexplored.

The gate‐opening behavior of flexible PCPs is highly advantageous for gas storage applications because it allows significantly higher working capacities within a narrow pressure range than those achieved by conventional Langmuir‐type adsorbents [16]. Considerable progress has been made in tuning gate‐opening pressures through solid‐solution approaches and crystal‐engineering strategies [17, 18, 19]. However, simultaneously enhancing framework flexibility and storage capacity remains a significant challenge. Stronger host–guest interactions are generally required to stabilize expanded framework structures; however, excessively strong interactions increase the energy required for guest release. Consequently, the design of flexible PCPs capable of achieving large pore expansion requires a delicate balance between framework deformation and adsorption energetics.

**FIGURE 1:**
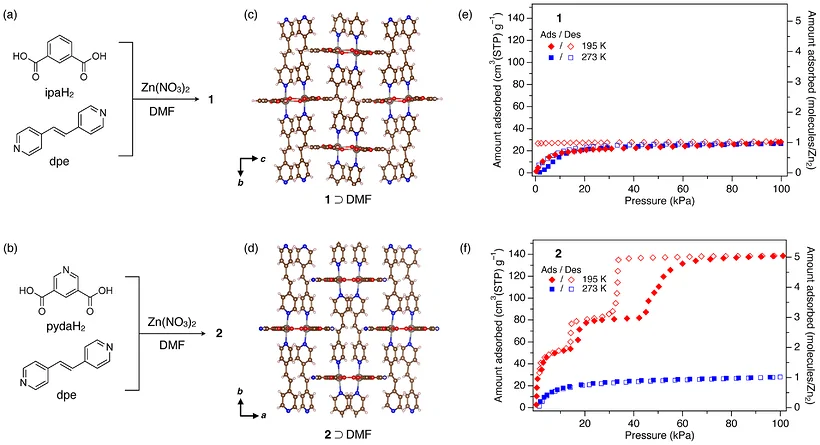
Synthetic schemes of (a) **1** and (b) **2**. Crystal structures of as‐synthesized phases for (c) **1** (**1**⊃DMF) and (d) **2** (**2**⊃DMF), determined by SXRD measurements at 93 K (Table S1). Carbon, gray; nitrogen, blue; oxygen, red; zinc, green. DMF molecules residing in the pores are omitted for clarity because their positions could not be reliably determined due to severe disorder. Acetylene adsorption (closed symbols) and desorption (open symbols) isotherms for (e) **1** and (f) **2**, measured at 195 K (red rhombuses) and 273 K (blue squares).

In this context, identifying rational methods to stabilize expanded framework phases and unlock hidden structural flexibility represents an important challenge in the development of responsive porous materials. Herein, we demonstrate that a subtle atomic‐level modification of a framework ligand can dramatically alter the adsorption behavior and structural flexibility of a PCP. We focused on CID (coordination polymer with interdigitated framework)‐type flexible PCPs [18, 19, 20, 21, 22, 23] and synthesized the framework [Zn(ipa)(dpe)]*_n_* (ipa = isophthalate, dpe = 1,2‐di(4‐pyridyl)ethylene), referred to as **1** (Figure 1). Upon acetylene sorption, however, **1** exhibited a featureless isotherm with no observable GO/GC behavior and a moderate adsorption capacity of approximately 30 cm^3^ g^−1^ (STP) at 195 K. In contrast, we identified another isostructural CID framework using pyridine‐3,5‐dicarboxylic acid (pydaH_2_) as a ligand instead of ipaH_2_, namely [Zn(pyda)(dpe)]*_n_* (referred to as **2**) (Figure 1), which displayed multistep GO‐type isotherms with a significantly enhanced adsorption capacity of approximately 140 cm^3^ g^−1^ (STP) at 195 K.

The incorporation of pyda, which features a free pyridinic nitrogen as a hydrogen bond acceptor, not only helps **2** selectively capture acetylene but also promotes further acetylene uptake, triggering an extra deformation mode that increases the pore volume of **2**. This results in a 276% increase in pore volume compared to **1** that has no hydrogen bonding sites. Further, **2** exhibits a four‐step adsorption process for acetylene but limited adsorption steps and capacity for ethane and ethylene. The acetylene‐specific deformation mechanisms were elucidated through single‐crystal and powder X‐ray diffraction analyses. This study provides valuable insights into designing new flexible PCPs, where incorporating directional hydrogen bonding motifs into the framework can significantly enhance storage capacities by inducing inherent structural expansion.

## 2 Results and Discussion

### 2.1 Synthesis and Structure of 1 and 2

**FIGURE 2:**
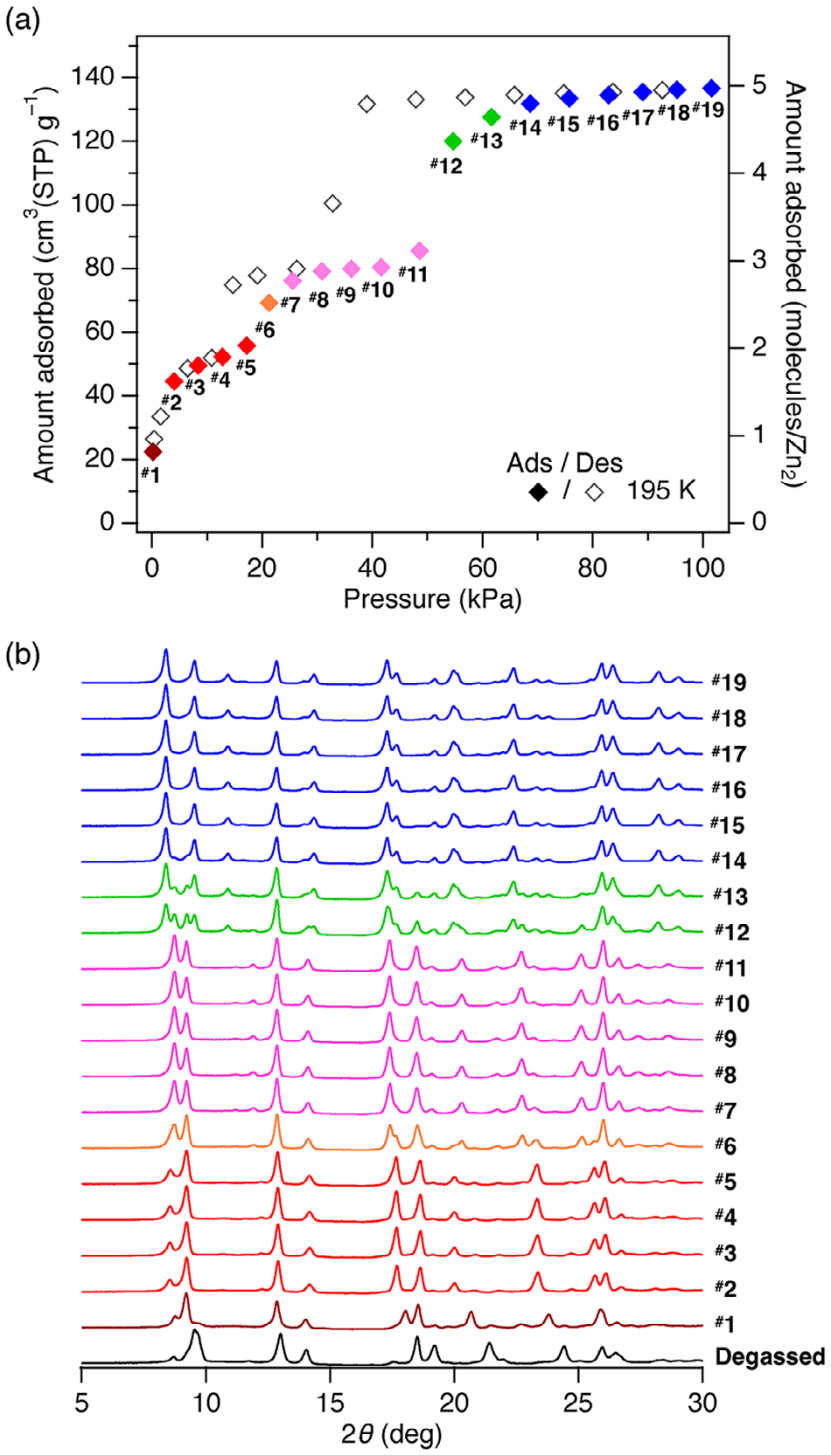
In situ acetylene adsorption and PXRD measurements for **2**. (a) Acetylene adsorption (closed symbols) and desorption (open symbols) isotherms of **2** measured at 195 K. (b) In situ PXRD patterns of **2** recorded at each equilibrium pressure point (^#^1–^#^19) along the isotherm shown in panel (a), corresponding to the stepwise structural transformations during gas adsorption.

Single crystals of **1** and **2** were synthesized using a slow diffusion method at 80°C, employing layered DMF solutions of Zn(NO_3_)_2_ with the pillar and layer ligands dpe and ipaH_2_ for **1** (Figure 1a), and dpe and pydaH_2_ for **2**, respectively (Figure 1b), see Section 4). These reactions yielded colorless crystals in the solvated forms, denoted as **1**⊃DMF and **2**⊃DMF. Single‐crystal X‐ray diffraction (SXRD) analysis confirmed a typical CID‐type structure in the expanded form for both **1** (Figure 1c) and **2** (Figure 1d) [21]. Based on the SXRD data, the guest‐accessible void spaces (void fraction) of **1**⊃DMF and **2**⊃DMF were measured to be 19.0% and 18.0% per unit cell, respectively (Table S1). The close similarity of those structural features is consistent with the minor structural differences between the ipa and pyda layer ligands.

The powder samples of **1** and **2** were synthesized according to the above single‐crystal synthetic method (see Section 4). The powder X‐ray diffraction patterns of the prepared powder samples showed diffraction patterns identical to those simulated from the SXRD data (Figure S1), confirming the successful large‐scale synthesis of both PCPs in a powdery form.

### 2.2 Gas Sorption Properties and Structure Changes

Acetylene gas adsorption measurements were performed for **1** and **2** at 195 K and 273 K. At 195 K, **1** exhibited a typical Langmuir‐type adsorption isotherm, with an adsorption capacity of 27.8 mL g^−1^ at 100 kPa (Figure 1e; Figure S2). This corresponds to the incorporation of one acetylene molecule per unit cell, or 0.5 mol of acetylene per mol of Zn^2+^ within the framework. In contrast, **2** displayed distinctly different adsorption behavior under the same conditions (Figure 1f). The acetylene adsorption isotherm of **2** at 195 K exhibited distinct adsorption increases characteristic of GO behavior at ∼3 kPa, 18 kPa, and 45 kPa, with four well‐defined adsorption stages. The first GO step occurred in a low‐pressure range (2–5 kPa) and transitioned rapidly to the second step, reaching Stage 2 (Figure S2). The adsorption capacities at each adsorption stage corresponded to 0.5, 1.0, 1.5, and 2.5 mol of acetylene per Zn, equivalent to the stepwise incorporation of one, two, three, and five acetylene molecules per unit cell (i.e., per Zn_2_), respectively (Figure 1f; Figure S2). This stepwise adsorption process was reversible, as the desorption isotherm revealed similar stepwise acetylene release (Figure 1f).

Such multi‐step gas adsorption behavior, exceeding four GO/GC steps, is exceptionally rare [24]. Notably, the acetylene storage capacity of **2** was five times greater than that of **1**, emphasizing the significant impact of a minor structural modification to the layer ligand on adsorption behavior and overall capacity. To investigate the structural deformation of **2** during acetylene adsorption, we conducted in situ powder X‐ray diffraction (PXRD) measurements combined with gas adsorption analysis at 195 K (Figure 2 and see Section 4). PXRD patterns were recorded at each equilibrium pressure point along the acetylene adsorption isotherm. The resulting profiles exhibited distinct changes at GO pressure, providing clear evidence of a four‐step structural deformation process. Thus, the multistep GO adsorption is closely tied to the structural deformation of the framework, underscoring the importance of precise structural design in flexible PCPs.

At a higher temperature of 273 K, however, both **1** and **2** exhibited Langmuir‐type adsorption isotherms, with adsorption limited to the first stage (one acetylene per unit cell) (Figure 1e,f). The unique stepwise adsorption behavior observed for **2** at 195 K was absent at 273 K, likely because the adsorption energy at higher temperatures was insufficient to overcome the deformation energy required for structural transformations [7].

**FIGURE 3:**
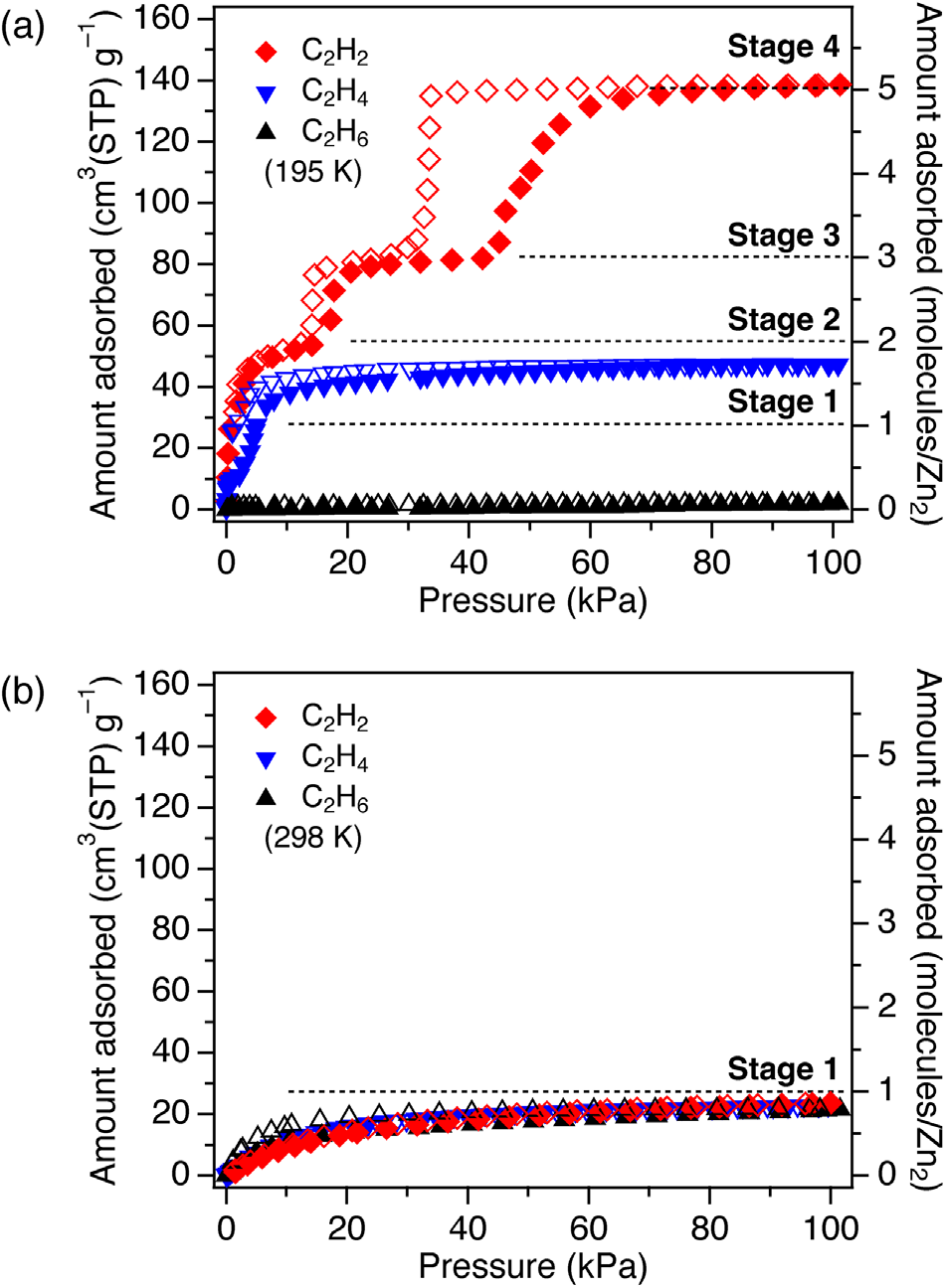
Adsorption (closed symbols) and desorption (open symbols) isotherms of acetylene (red rhombuses), ethylene (blue down triangles), and ethane (black up triangles), measured at (a) 195 K and (b) 298 K.

Interestingly, the structural flexibility of **2** was found to vary significantly depending on the type of gas. Adsorption measurements at 195 K with other C_2_ hydrocarbons, ethylene and ethane, revealed distinct behaviors (Figure 3a). For ethylene, **2** exhibited adsorption up to Stage 2 (two ethylene molecules per unit cell) but did not proceed to further adsorption or GO behavior. In contrast, ethane showed no adsorption and was entirely excluded from the framework. This variation in adsorption behavior among C_2_ hydrocarbons is likely due to differences in their acidity and their ability to engage in π interactions, both of which influence the strength of host–guest interactions. Hydrocarbons with more acidic hydrogen atoms interact more strongly with the pyridine ligand (pyda) via hydrogen bonding, leading to higher affinity. These interactions contribute to the stabilization of the expanded framework phases and thereby facilitate subsequent adsorption‐induced structural transitions. The results indicate that introducing Lewis basic ligands into the framework of flexible PCPs can effectively achieve exceptional discrimination among C_2_ hydrocarbons. Furthermore, the observed adsorption selectivity suggests that host–guest π interactions play complementary roles in stabilizing the adsorbed phases, providing important insight into the unique multistep gate‐opening behavior of **2** (*vide infra*).

At 298 K, however, the C_2_ hydrocarbon selectivity of **2** was lost, and all three gases displayed nearly identical Langmuir‐type isotherms (Figure 3b). Notably, even ethane, which was not adsorbed at 195 K, exhibited adsorption at 298 K up to Stage 1. These results suggest that the ability of **2** to differentiate between C_2_ hydrocarbons is temperature‐dependent. PXRD measurements under vacuum at varying temperatures revealed structural changes in degassed **2** with increasing temperature (Figure S3). Specifically, a diffraction peak corresponding to the (010) plane shifted from 9.6° to 9.3° as the temperature increased from 80 to 330 K, indicating expansion of the unit cell. SXRD analysis of degassed **2** further confirmed unit cell volume expansion from 897.3 to 964.7 Å^3^ between 123 and 298 K (Figure S4 and Table S1). Examination of the structural changes revealed that with increasing temperature, the angle between the layer ligand (pyda) and the pillar ligand (dpe) approached perpendicular to each other, accompanied by an increase in void fraction (Figure S4). This structural adjustment at higher temperatures resulted in pore opening at the initial degassed state, enabling uniform adsorption of all C_2_ hydrocarbons and a consequent loss of selectivity.

### 2.3 Structure Analysis on Each Acetylene‐Adsorbed Phase

**FIGURE 4:**
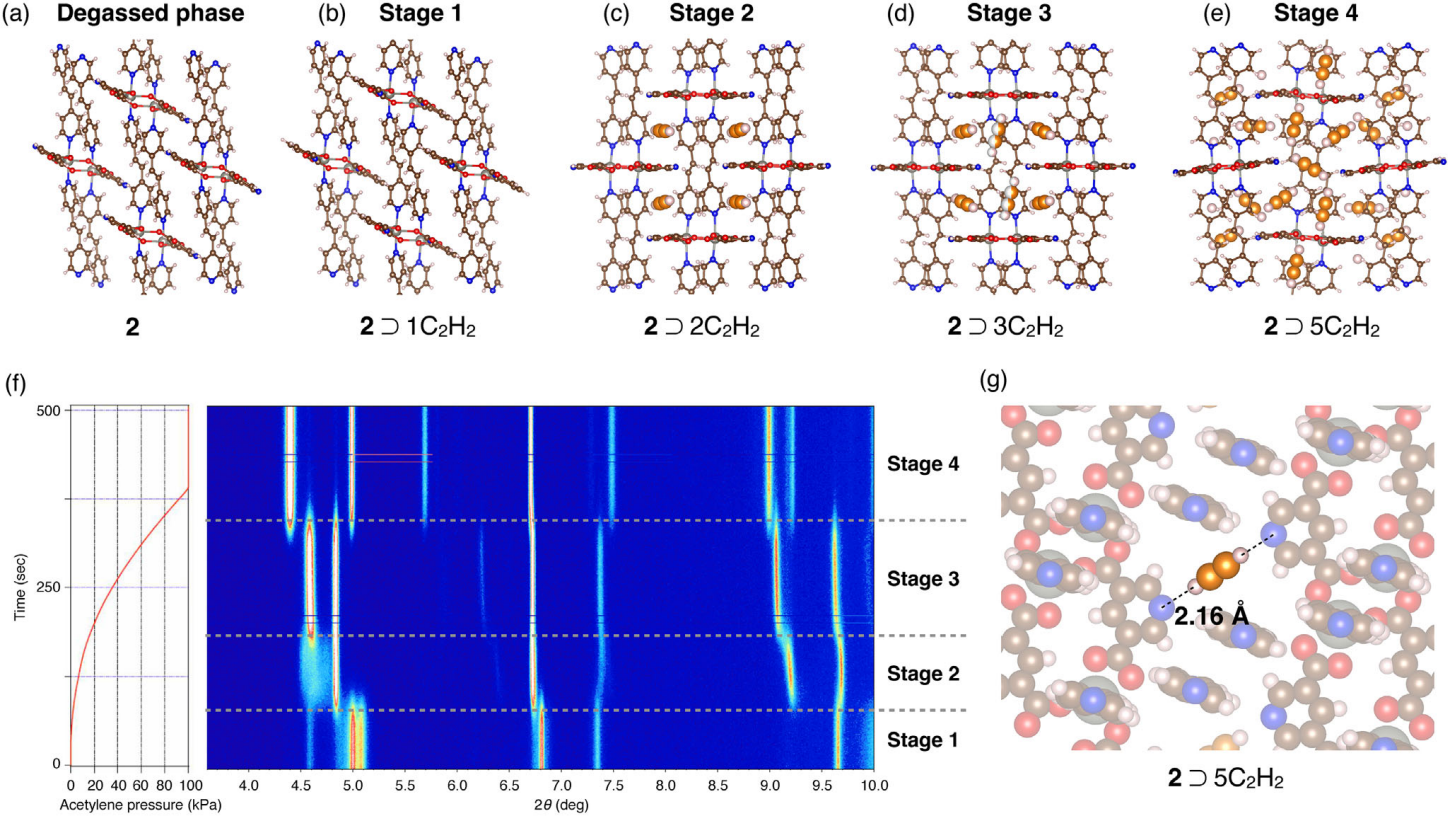
(a–e) Crystal structures of **2** in the (a) degassed phase, (b) Stage 1, (c) Stage 2, (d) Stage 3, and (e) Stage 4, as determined by SXRD and SR‐PXRD analyses. Detailed measurement conditions and crystallographic parameters are summarized in Tables S1 and S2. In panel (b), the adsorbed acetylene molecules in Stage 1 are not shown because their positions could not be reliably determined owing to severe disorder. (f) SR‐PXRD patterns collected under increasing acetylene pressure at 195 K, clearly showing distinct stepwise structural transitions from Stage 1 to Stage 4. (g) Crystal structure of **2** at Stage 4, with five acetylene molecules per unit cell. One acetylene molecule is captured at Site 4, positioned between two pyda ligands via hydrogen bonding. This hydrogen‐bonded interaction stabilizes the expanded framework and facilitates the unlocking of additional pore volume for further acetylene adsorption.

To elucidate the structural deformation mechanism of **2** during acetylene adsorption, we employed synchrotron radiation (SR) SXRD and powder X‐ray diffraction (SR‐PXRD) to determine the crystal structure at the degassed phase and each acetylene adsorption stage (Figure 4a–e). A single crystal of **2** was placed in a glass capillary and activated in situ, allowing for the SR‐SXRD analysis at the degassed phase (Figure 4a; Table S1). In the same manner, an activated single crystal was sealed with acetylene gas at 5 kPa, and SXRD analysis was performed at 195 K (see Section 4). The refined SXRD structure successfully provided the crystal structure corresponding to Stage 1 (Figure 4b; Table S1). The PXRD pattern simulated from the SXRD data matched the diffraction pattern observed for Stage 1 in the in situ PXRD/gas adsorption experiments (Figure S5). Although the position of the adsorbed acetylene could not be identified due to severe disorder, the expanded framework structure showed sufficient void space to accommodate one acetylene molecule per unit cell. This void space, located adjacent to one side of the layer ligand (pyda), is referred to as Site 1 (Figure S6). At Stage 1, the void fraction of **2** was determined to be 8.4% (Table S1).

Further analysis of the expanded structures of **2** was conducted using SR‐PXRD under controlled acetylene pressures (see Section 4). Acetylene gas was introduced at varying pressures into a capillary containing powdered **2**, and SR‐PXRD measurements were performed to monitor the structural transitions (Figure 4f). While the transformation from the degassed state to Stage 1 could not be captured due to the extremely low and narrow pressure range, successive structural transitions from Stage 2 to Stage 4 were observed as the acetylene pressure increased (Figure 4f). At each GO pressure, **2** transitioned instantaneously to the next step without passing through any intermediate structures (like breathing effect) [25, 26], indicating phase‐like structural transitions characteristic of gate‐opening adsorption in flexible PCPs [7].

Wide‐angle SR‐PXRD measurements at each step enabled structural analysis using the Rietveld–maximum entropy method (MEM), which revealed the detailed acetylene adsorption configurations. At Stage 2, the structure of **2** accommodated two acetylene molecules per unit cell (one acetylene per Zn) (Figures S7 and S8, Tables S2 and S3). The void fraction at Stage 2 was determined to be 21.6%, representing a 257% expansion compared to Stage 1 (Table S2). A new adsorption site, Site 2, was created adjacent to the layer ligand (pyda) due to a shift in the relative positions of the pillar ligands (dpe) (Figure S6). Acetylene molecules were captured at Site 1 and Site 2, positioned on opposite sides of the layer ligand. The orientation of the layer ligand at Stage 2 was nearly perpendicular (90°) to the pillar ligands, resembling the structure of the solvated crystal containing DMF molecules (**2**⊃DMF) (Figure 1d). This suggests that the Stage 2 structure represents the most stabilized and relaxed configuration of **2**, consistent with the observation that **2** adsorbs ethylene only up to Stage 2 under similar adsorption conditions (Figure 3a).

At Stage 3, **2** exhibited a more complex adsorption structure. The void fraction increased slightly to 27.2%, representing a modest 126% expansion compared to Stage 2 (Figures S9 and S10, Tables S2 and S4). Three acetylene molecules were identified per unit cell: two were located at Site 1 and Site 2, as in Stage 2, while a third acetylene molecule was observed at a newly formed site, Site 3, positioned between Site 1 and Site 2, with an occupancy of 50% (Figure S6).

At the fourth step, **2** underwent a significant structural transformation, with an increased void fraction of 31.6%, representing a 116% expansion from Stage 3, forming the final Stage 4 (Figures S11 and S12, Tables S2 and S5). The total void volume expansion reached 276% from Stage 1. An additional acetylene molecule was captured at a newly formed site, Site 4, located between opposing pyda ligands via hydrogen bonding with their nitrogen atoms (Figure S6). This interaction caused further expansion of the framework. Moreover, Stage 4 allowed the acetylene molecule at Site 3 to fully occupy the site, resulting in the adsorption of a total of five acetylene molecules per unit cell (2.5 acetylene per Zn). The distance between the acetylene hydrogen at Site 4 and the nitrogen atom of the pyda ligand was measured as 2.16 Å (Figure 4g; Figure S13), confirming strong hydrogen‐bonding interactions [8, 9]. This interaction stabilized the expanded framework and induced an additional phase with significant structural deformation, facilitating further acetylene adsorption.

Additionally, we investigated the π interactions within the framework to gain further mechanistic insight into the gate‐opening behavior and C_2_ adsorption selectivity of **2**. Structural analysis of the acetylene‐loaded phases at Stages 2 and 3 (195 K) revealed host–guest π interactions between the pyda ligands and the adsorbed acetylene molecules (Figures S8 and S10), suggesting that these interactions contribute to stabilization of the expanded framework phases and enhance C_2_ adsorption selectivity. Although the positions of the acetylene molecules in Stage 1 could not be determined because of severe disorder, similar host–guest π interactions are expected to operate in this phase. Therefore, the absence of π orbitals in ethane likely prevents the formation of sufficiently strong host–guest interactions to initiate the first gate‐opening transition, resulting in negligible adsorption at low temperatures (Figure 3a). In the Stage 3 structure, one of the three acetylene molecules appears to be stabilized by π interactions with the dpe ligand (Figure S10).

Interestingly, the Stage 4 structure reveals a distinct stabilization mechanism. An acetylene molecule inserted between two pyda ligands is strongly stabilized through hydrogen‐bonding interactions, whereas the remaining four acetylene molecules appear to be accommodated primarily through weaker van der Waals interactions (Figure S12). These observations suggest that hydrogen bonding plays a crucial role in stabilizing the highly expanded framework phase, thereby enabling further framework deformation and creating additional pore volume for subsequent acetylene adsorption.

These results demonstrate that incorporating hydrogen‐bonding groups into PCP structures is a highly effective strategy for enhancing structural flexibility and enabling additional framework expansion in flexible PCPs. The findings highlight the potential of hydrogen bonding to unlock new deformation modes, significantly improving gas storage capacities.

## 3 Conclusions

In this study, we investigated CID‐type flexible PCPs, focusing on the significant changes in gas adsorption behavior induced by subtle chemical modifications to the ligands. By replacing the layer ligand of the CID framework from the conventional isophthalic acid (ipaH_2_) to pyridine‐3,5‐dicarboxylic acid (pydaH_2_), we observed a remarkable enhancement in acetylene adsorption capacity. The nitrogen atom on the pyda ligand functions as a hydrogen bond acceptor, facilitating interactions with acetylene molecules and enabling their capture within the framework. This interaction activates the latent structural flexibility of the CID‐type PCP, resulting in a 276% pore expansion rate and a fivefold increase in acetylene storage capacity compared to the ipa‐based PCPs. Moreover, despite the minimal chemical differences between the ligands, the substitution also altered the framework's flexibility and selectivity toward other C_2_ gases, enabling discriminative gate‐opening behavior for acetylene while suppressing adsorption of ethylene and ethane.

These findings provide valuable insights into how small structural variations in ligands can lead to significant differences in gas storage performance. Additionally, they highlight the potential of hydrogen‐bonding ligands to stabilize and unlock hidden structural phases in PCPs, thereby achieving enhanced storage capacities. This work offers an important guideline for the future structural design and optimization of flexible PCPs.

## 4 Experimental Section

### 4.1 Materials

All reagents and solvents used in this study were obtained from Nacalai Tesque, Wako Pure Chemicals, and Tokyo Chemical Industry (TCI), unless otherwise noted. All chemicals were used as received without any further purification.

### 4.2 Single Crystal Preparation

Single crystals of **1** and **2** were prepared by a slow diffusion method. Two DMF solutions containing Zn(NO_3_)_2_ and the corresponding ligands were used: dpe and ipaH_2_ for **1**, and dpe and pydaH_2_ for **2**. These solutions were carefully layered in a glass tube. The molar ratio of metal to layer ligand to pillar ligand was maintained at 1:1:1, with a concentration of 15 mmol/L. The layered tubes were placed in a thermostated oven at 80°C and left undisturbed for several days. This procedure resulted in the formation of colorless block‐shaped single crystals. The products were identified as **1**⊃DMF ([Zn(ipa)(dpe)]*_n_*, 0.1 × 0.05 × 0.5 mm^3^) and **2**⊃DMF ([Zn(pyda)(dpe)]*_n_*, 0.1 × 0.05 × 0.2 mm^3^).

### 4.3 Powder Sample Preparation

Powdered **1** was synthesized as follows. In a round‐bottom flask, Zn(NO_3_)_2_ (1 eq., 5 mmol), ipaH_2_ (1 eq., 5 mmol), and dpe (1 eq., 5 mmol) were dissolved in DMF (total volume: 200 mL) at 25°C. The reaction mixture was heated under stirring in an oil bath at 100°C for 24 h. The resulting white precipitate was collected by centrifugation at 3,500 rpm (2,300 × g) for 2 min, washed thoroughly with excess DMF, and centrifuged again under the same conditions. The final product was dried under reduced pressure at 25°C for several days to afford a white powder (yield: 883 mg).

Powdered **2** was prepared similarly. Zn(NO_3_)_2_ (1 eq., 7 mmol), pydaH_2_ (1 eq., 7 mmol), and dpe (1 eq., 7 mmol) were dissolved in DMF (total volume: 300 mL) at 25°C. The solution was stirred and heated in an oil bath at 80°C for 24 h. The resulting white precipitate was isolated by centrifugation at 3,500 rpm (2,300 × g) for 2 min, washed with excess DMF, and subjected to a second centrifugation step under identical conditions. The collected white powder was dried under reduced pressure at 25°C for several days, yielding the final product (1.11 g).

### 4.4 Gas Adsorption Measurement

Gas adsorption isotherms were measured using automatic volumetric adsorption analyzers, BELSORP‐max and BELSORP‐mini‐II (MicrotracBEL Corp., Japan). Temperature control for the BELSORP‐max system was achieved using a custom‐made cryostat. The BELSORP‐mini‐II system employed a cooling bath (liquid nitrogen or dry ice/isopropanol mixture, depending on the measurement temperature). Prior to measurement, the samples were pre‐dried and loaded into the sample cell, followed by evacuation under vacuum at 150°C for 12 h. Adsorption isotherms were then recorded by monitoring pressure changes and calculating the amount of gas adsorbed based on the pressure drop at equilibrium. Equilibrium at each measurement point was defined as a pressure change of less than 0.05% over 300 s. The dead volume of the sample cell was determined using helium. All gases used for the measurements were of high purity, over 99.999%.

### 4.5 In Situ PXRD/Gas Sorption Measurement

In situ PXRD combined with gas sorption measurements was conducted using a BELSORP‐18PLUS automated volumetric adsorption analyzer (MicrotracBEL Corp., Japan) equipped with a custom‐made cryostat temperature control system. The PXRD measurements were performed using a Rigaku SmartLab diffractometer with Cu Kα radiation, which was directly coupled to the BELSORP‐18PLUS apparatus. The two systems were synchronized to allow PXRD pattern acquisition at each equilibrium point along the gas sorption isotherm, enabling simultaneous monitoring of structural transformations and gas uptake.

Prior to measurement, the samples were activated externally by heating at 150°C for 11 h under vacuum, followed by an additional internal activation step at 150°C for 2 h within the measurement system. This procedure ensured complete removal of guest molecules and reproducible framework activation for accurate sorption and structural analysis.

### 4.6 SXRD Measurement and Structure Refinement

SXRD measurements were conducted at 93 K using a Rigaku XtaLab P200 diffractometer equipped with a Dectris Pilatus 200K CCD detector and a MicroMax–007 HF/VariMax rotating anode X‐ray generator with confocal monochromated Mo Kα radiation. Crystal structures were solved by direct methods and refined by full‐matrix least‐squares refinement using SHELXL 2017/1. In cases where significant disorder was present, solvents and guest molecules included in the crystalline lattices were removed using the SQUEEZE routine implemented in the PLATON software package. The hydrogen atoms were added using the riding model.

The as‐synthesized single crystals, **1**⊃DMF and **2**⊃DMF, were coated with Paratone oil and mounted on a MicroMount (MiteGen), followed by analysis at 123 and 93 K, respectively, under a nitrogen gas flow.

For degassed‐state analysis, a single crystal of **2**⊃DMF was placed in a 3‐cm‐long borosilicate capillary (0.3 mm inner diameter, 0.01 mm wall thickness). The capillary was heated at 120°C and evacuated under dynamic vacuum (≤ 10^−2^ Pa) using a turbo‐molecular pump for 12 h, then sealed under vacuum. The sealed capillary was cooled to 123 or 298 K at a controlled rate of 1°C/min using a nitrogen gas flow. SXRD data were collected at 123 or 298 K to determine the crystal structure in the degassed state.

For SXRD analysis of the C_2_H_2_‐loaded state, the SR X‐ray source at the beamline BL02B1 of SPring‐8, Japan, was used. The crystal was heated and evacuated using the identical conditions within the glass capillary, and then C_2_H_2_ gas was introduced until the internal pressure reached 5 kPa at 25°C. The capillary was then sealed while maintaining this pressure. After sealing, the sample was cooled to 195 K at a rate of 1°C/min using nitrogen gas flow, and SXRD data were collected at this temperature to analyze the C_2_H_2_‐loaded structure (**2**⊃1C_2_H_2_).

Crystallographic data are summarized in Table S1. Crystal structures in CIF format have been deposited with the Cambridge Crystallographic Data Centre (CCDC) under deposition numbers CCDC 2496649–2496653. These data can be obtained free of charge from the CCDC via www.ccdc.cam.ac.uk/data_request/cif or by contacting the Cambridge Crystallographic Data Centre, 12 Union Road, Cambridge CB2 1EZ, United Kingdom.

### 4.7 SR‐PXRD Measurements and Structure Refinement

SR‐PXRD measurements were conducted at beamline BL02B2 of SPring‐8, Japan, using a Debye–Scherrer diffractometer equipped with MYTHEN one‐dimensional semiconductor detectors [27]. In situ gas adsorption experiments were carried out with a Remote Gas‐ and Vapor‐Pressure Control (RGVPC) system [28], enabling real‐time control of gas pressure and sample temperature. Temperature was regulated by a nitrogen‐stream cooling device. Powder samples were loaded into borosilicate capillaries (0.5 mm i.d.) and packed to a length of ∼2 mm. To monitor structural changes during acetylene adsorption, continuous diffraction data (1 s per frame; 500 frames) were collected at 195 K under pressures ranging from 0 to 100 kPa after evacuating the sample at 423 K for ∼15 min. For detailed structure determination, long‐exposure measurements (900 s) were performed at 195 K under fixed acetylene pressures of 14, 40, and 100 kPa after confirming adsorption equilibrium.

Diffraction patterns were indexed using DICVOL [29] and Conograph [30], software, with DICVOL used for the adsorbed phases and Conograph for the degassed phase. Structure determination was employed by the MEM/Rietveld method, which combines conventional Rietveld refinement with MEM‐based electron density analysis [31, 32, 33] using ENIGMA software [34]. Rietveld refinement was first performed using a framework‐alone model based on guest‐free single‐crystal structures of **2**, followed by MEM analysis to visualize electron density. Acetylene molecules were then placed at electron density maxima in the pores, and the model was modified iteratively until consistency was achieved between the refined structural model and the MEM‐derived electron density. Final Rietveld refinements yielded the optimized acetylene‐adsorbed structures for **2**⊃2C_2_H_2_, **2**⊃3C_2_H_2_, and **2**⊃5C_2_H_2_ (Tables S3–S5). Crystallographic data are summarized in Table S2. Crystal structures in CIF format have been deposited with CCDC under deposition numbers CCDC 2558775 (**2**⊃2C_2_H_2_), 2558972 (**2**⊃3C_2_H_2_), and 2558977 (**2**⊃5C_2_H_2_).

## Funding

This work was funded by the KAKENHI JSPS: JP25000007, JP18H05262, JP20H02575, JP20H04466, and JP22H05005.

## Conflicts of Interest

C.L. is a research project manager at Air Liquide Innovation Campus in Paris, France. S.K. was partially funded by Air Liquide in the frame of a collaborative research agreement between Air Liquide and Kyoto University. The other authors do not declare any competing interests.

## Supporting information




**Supporting File**: smll74777‐sup‐0001‐SuppMat.pdf.

## Data Availability

X‐ray crystallographic data have been deposited at the CCDC (http://www.ccdc.cam.ac.uk/) under CCDC nos. 2496649, 2496650, 2496651, 2496652, 2496653, 2558775, 2558972, and 2558977. All other data supporting the findings of this study are available within the article and the Supplementary Information

## References

[1] A. G. Slater and A. I. Cooper , “Function‐Led Design of New Porous Materials,” Science 348 (2015): aaa8075. 10.1126/science.aaa8075 26023142

[2] M. L. Foo , R. Matsuda , and S. Kitagawa , “Functional Hybrid Porous Coordination Polymers,” Chemistry of Materials 26 (2014): 310–322.

[3] H. Lyu , Z. Ji , S. Wuttke , and O. M. Yaghi , “Digital Reticular Chemistry,” Chemistry 6 (2020): 2219–2241.

[4] M. Eddaoudi , J. Kim , N. Rosi , et al., “Systematic Design of Pore Size and Functionality in Isoreticular MOFs and Their Application in Methane Storage,” Science 295 (2002): 469–472. 10.1126/science.1067208 11799235

[5] G. Hu , Q. Liu , and H. Deng , “Space Exploration of Metal–Organic Frameworks in the Mesopore Regime,” Accounts of Chemical Research 58 (2025): 73–86. 10.1021/acs.accounts.4c00633 39668693

[6] S. Kitagawa , R. Kitaura , and S. Noro , “Functional Porous Coordination Polymers,” Angewandte Chemie International Edition 43 (2004): 2334–2375. 10.1002/anie.200300610 15114565

[7] S. Krause , N. Hosono , and S. Kitagawa , “Chemistry of Soft Porous Crystals: Structural Dynamics and Gas Adsorption Properties,” Angewandte Chemie International Edition 59 (2020): 15325–15341. 10.1002/anie.202004535 32458545

[8] R. Matsuda , R. Kitaura , S. Kitagawa , et al., “Highly Controlled Acetylene Accommodation in a Metal–organic Microporous Material,” Nature 436 (2005): 238–241. 10.1038/nature03852 16015325

[9] Y. Gu , J. Zheng , K. Otake , et al., “Host–Guest Interaction Modulation in Porous Coordination Polymers for Inverse Selective CO_2_/C_2_H_2_ Separation,” Angewandte Chemie International Edition 60 (2021): 11688–11694. 10.1002/anie.202016673 33594724

[10] Y. Ma , R. Matsuda , H. Sato , et al., “A Convenient Strategy for Designing a Soft Nanospace: An Atomic Exchange in a Ligand with Isostructural Frameworks,” Journal of the American Chemical Society 137 (2015): 15825–15832. 10.1021/jacs.5b09666 26592095

[11] Z. Niu , X. Cui , T. Pham , et al., “A MOF‐Based Ultra‐Strong Acetylene Nano‐Trap for Highly Efficient C_2_H_2_/CO_2_ Separation,” Angewandte Chemie International Edition 60 (2021): 5283–5288. 10.1002/anie.202016225 33403811

[12] X. Cui , K. Chen , H. Xing , et al., “Pore Chemistry and Size Control in Hybrid Porous Materials for Acetylene Capture from Ethylene,” Science 353 (2016): 141–144. 10.1126/science.aaf2458 27198674

[13] R. Yang , Y. Wang , J.‐W. Cao , et al., “Hydrogen Bond Unlocking‐Driven Pore Structure Control for Shifting Multi‐Component Gas Separation Function,” Nature Communications 15 (2024): 804. 10.1038/s41467-024-45081-w PMC10821866 38280865

[14] Y. Ye , S. Xian , H. Cui , et al., “Metal–Organic Framework Based Hydrogen‐Bonding Nanotrap for Efficient Acetylene Storage and Separation,” Journal of the American Chemical Society 144 (2022): 1681–1689. 10.1021/jacs.1c10620 34965123

[15] J. Cao , T. Zhang , Y. Liu , et al., “Precise C_2_H_2_ Adsorption Affinity Modulation by Nitrogen Functionalization in Isostructural Coordination Networks,” Small 21 (2025): 2501924. 10.1002/smll.202501924 40033866

[16] S. Hiraide , Y. Sakanaka , H. Kajiro , S. Kawaguchi , M. T. Miyahara , and H. Tanaka , “High‐Throughput Gas Separation by Flexible Metal–organic Frameworks with Fast Gating and Thermal Management Capabilities,” Nature Communications 11 (2020): 3867. 10.1038/s41467-020-17625-3 PMC7400644 32747638

[17] M. Bonneau , C. Lavenn , J.‐J. Zheng , et al., “Tunable Acetylene Sorption by Flexible Catenated Metal–organic Frameworks,” Nature Chemistry 14 (2022): 816–822. 10.1038/s41557-022-00928-x 35449219

[18] S. Horike , Y. Inubushi , T. Hori , T. Fukushima , and S. Kitagawa , “A Solid Solution Approach to 2D Coordination Polymers for CH_4_/CO_2_ and CH_4_/C_2_H_6_ Gas Separation: Equilibrium and Kinetic Studies,” Chemical Science 3 (2012): 116–120.

[19] T. Fukushima , S. Horike , Y. Inubushi , et al., “Solid Solutions of Soft Porous Coordination Polymers: Fine‐Tuning of Gas Adsorption Properties,” Angewandte Chemie International Edition 49 (2010): 4820–4824. 10.1002/anie.201000989 20491105

[20] D. Tanaka , K. Nakagawa , M. Higuchi , et al., “Kinetic Gate‐Opening Process in a Flexible Porous Coordination Polymer,” Angewandte Chemie International Edition 47 (2008): 3914–3918. 10.1002/anie.200705822 18404764

[21] S. Horike , D. Tanaka , K. Nakagawa , and S. Kitagawa , “Selective Guest Sorption in an Interdigitated Porous Framework with Hydrophobic Pore Surfaces,” Chemical Communications 43 (2007): 3395–3397. 10.1039/b703502k 18019509

[22] K. Nakagawa , D. Tanaka , S. Horike , S. Shimomura , M. Higuchi , and S. Kitagawa , “Enhanced Selectivity of CO_2_ from a Ternary Gas Mixture in an Interdigitated Porous Framework,” Chemical Communications 46 (2010): 4258. 10.1039/c0cc00027b 20461245

[23] H. Sato , R. Matsuda , M. H. Mir , and S. Kitagawa , “Photochemical Cycloaddition on the Pore Surface of a Porous Coordination Polymer Impacts the Sorption Behavior,” Chemical Communications 48 (2012): 7919–7921. 10.1039/c2cc31083j 22450780

[24] S.‐Q. Wang , X.‐Q. Meng , M. Vandichel , et al., “High Working Capacity Acetylene Storage at Ambient Temperature Enabled by a Switching Adsorbent Layered Material,” ACS Applied Materials & Interfaces 13 (2021): 23877–23883. 10.1021/acsami.1c06241 PMC8289182 33983706

[25] G. Férey and C. Serre , “Large Breathing Effects in Three‐Dimensional Porous Hybrid Matter: Facts, Analyses, Rules and Consequences,” Chemical Society Reviews 38 (2009): 1380–1399. 10.1039/b804302g 19384443

[26] F.‐X. Coudert , A. Boutin , A. H. Fuchs , and A. V. Neimark , “Adsorption Deformation and Structural Transitions in Metal–Organic Frameworks: From the Unit Cell to the Crystal,” The Journal of Physical Chemistry Letters 4 (2013): 3198–3205.

[27] S. Kawaguchi , M. Takemoto , K. Osaka , et al., “High‐Throughput Powder Diffraction Measurement System Consisting of Multiple MYTHEN Detectors at Beamline BL02B2 of SPring‐8,” Review of Scientific Instruments 88 (2017): 085111. 10.1063/1.4999454 28863664

[28] S. Kawaguchi , M. Takemoto , H. Tanaka , S. Hiraide , K. Sugimoto , and Y. Kubota , “Fast Continuous Measurement of Synchrotron Powder Diffraction Synchronized with Controlling Gas and Vapour Pressures at Beamline BL02B2 of SPring‐8,” Journal of Synchrotron Radiation 27 (2020): 616–624. 10.1107/S1600577520001599 PMC7285677 32381761

[29] A. Boultif and D. Louër , “Powder Pattern Indexing with the Dichotomy Method,” Journal of Applied Crystallography 37 (2004): 724–731.

[30] R. Oishi‐Tomiyasu , “Robust Powder Auto‐Indexing Using Many Peaks,” Journal of Applied Crystallography 47 (2014): 593–598.

[31] M. Takata , B. Umeda , E. Nishibori , et al., “Confirmation by X‐Ray Diffraction of the Endohedral Nature of the Metallofullerene Y@C_82_ ,” Nature 377 (1995): 46–49.

[32] M. Takata , E. Nishibori , and M. Sakata , “Charge Density Studies Utilizing Powder Diffraction and MEM. Exploring of High Tc Superconductors, C_60_ Superconductors and Manganites,” Zeitschrift für Kristallographie‐Crystalline Materials 216 (2001): 71–86.

[33] Y. Kubota , M. Takata , T. C. Kobayashi , and S. Kitagawa , “Observation of Gas Molecules Adsorbed in the Nanochannels of Porous Coordination Polymers by the in Situ Synchrotron Powder Diffraction Experiment and the MEM/Rietveld Charge Density Analysis,” Coordination Chemistry Reviews 251 (2007): 2510–2521.

[34] H. Tanaka , M. Takata , E. Nishibori , K. Kato , T. Iishi , and M. Sakata , “ENIGMA: Maximum‐Entropy Method Program Package for Huge Systems,” Journal of Applied Crystallography 35 (2002): 282–286.

