# Supplementary material for: Unlocking Extra Flexibility of Soft Porous Crystals via Hydrogen Bonding Interaction for Multistep Acetylene Sorption
Source: Small. 2026 Jul 25;22(53):e74777. doi: 10.1002/smll.74777 (PMC13592413; doi:10.1002/smll.74777)
Supplement: Supplementary file 1 [file SMLL-22-e74777-s001.pdf]

Supporting Information

Unlocking Extra Flexibility of Soft Porous Crystals via Hydrogen Bonding Interaction for Multistep Acetylene Sorption

Christophe Lavenn, Nobuhiko Hosono, Ken-ichi Otake, Susan Sen, Shinpei Kusaka, Tomoe Watanabe, Hirotaka Ashitani, Shogo Kawaguchi, Hiroki Ishibashi, Yoshiki Kubota,* Ryotaro Matsuda, and Susumu Kitagawa**

1. Supporting Figure

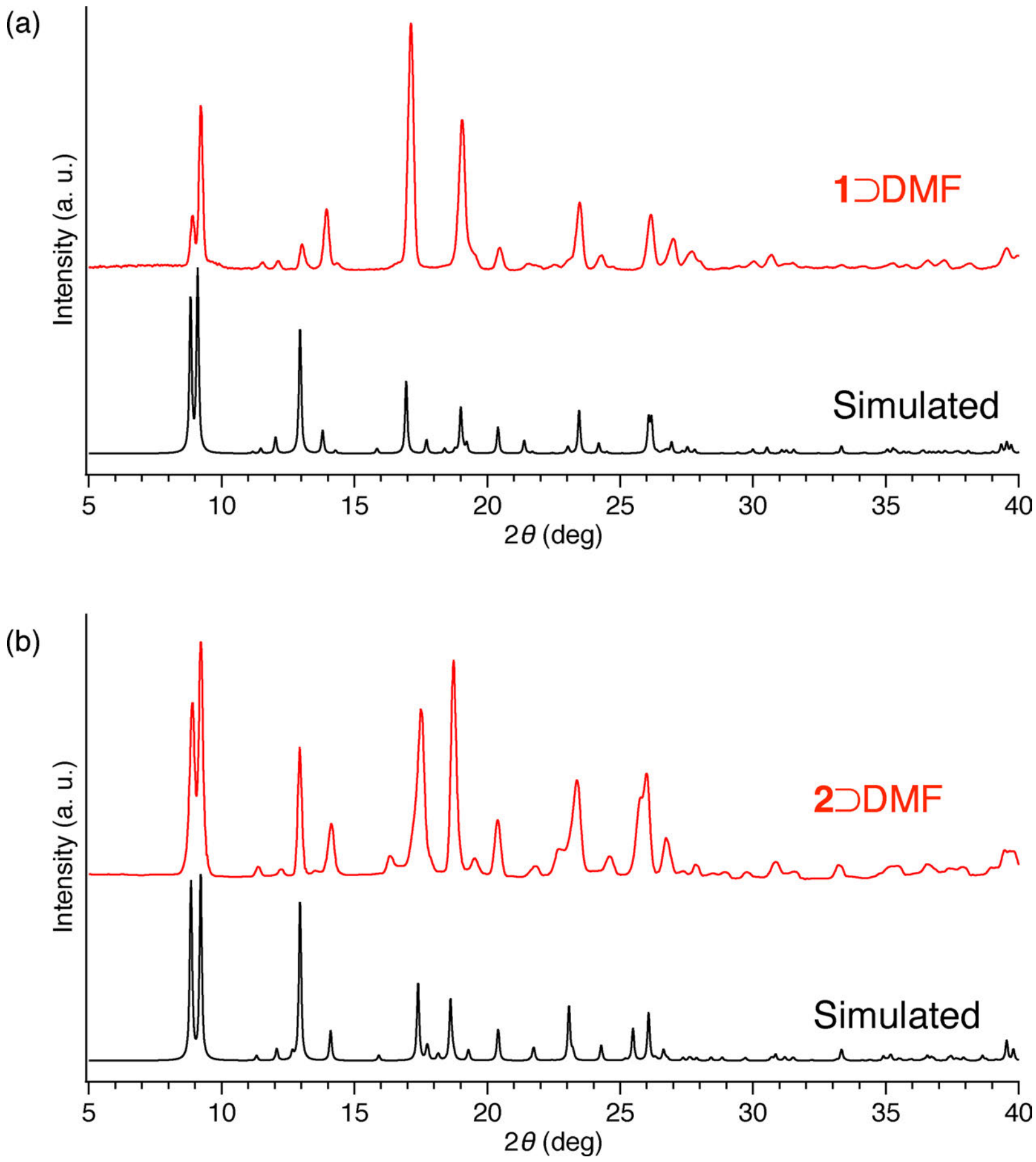

Figure S1. PXRD data of the powder samples of (a) **1** and (b) **2** in the solvated forms (as-synthesized in DMF). Simulated XRD patterns are shown as black traces.

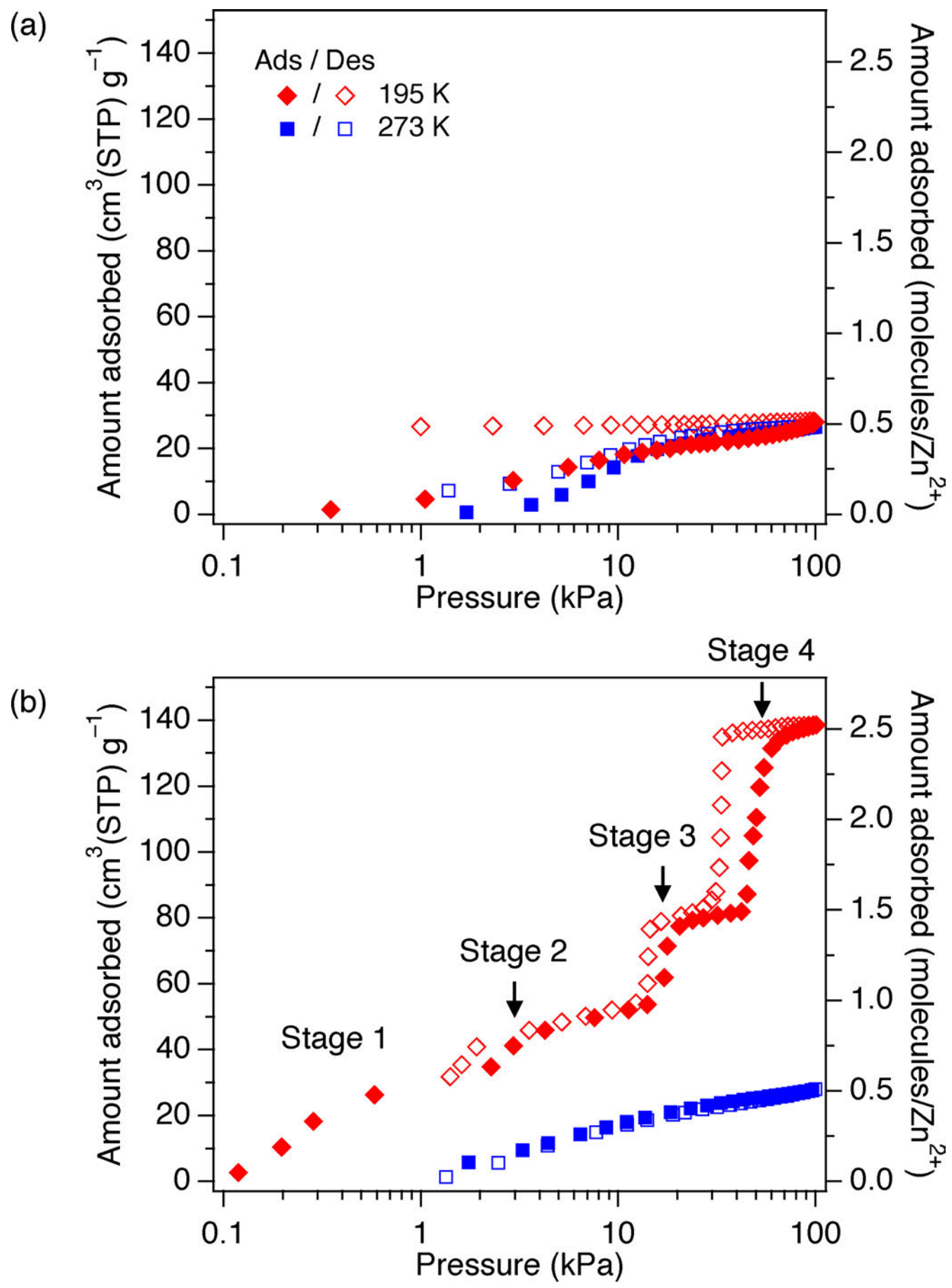

Figure S2. Semi-logarithmic C_2H_2 adsorption isotherms of (a) **1** and (b) **2**, measured at 195 K (red rhombuses) and 273 K (blue squares). The arrows in panel b indicate the GO pressures at ~3 kPa, 18 kPa, and 45 kPa.

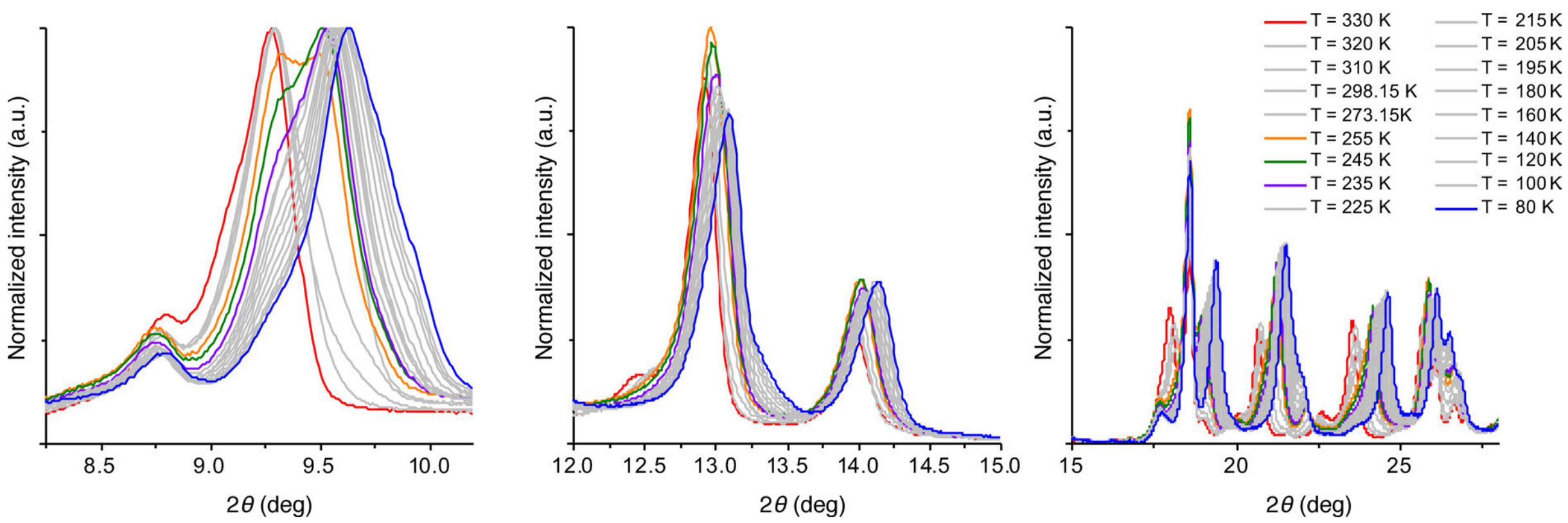

Figure S3. PXRD patterns of **2** recorded at varying temperatures, showing characteristic peak shifts associated with thermally induced structural changes.

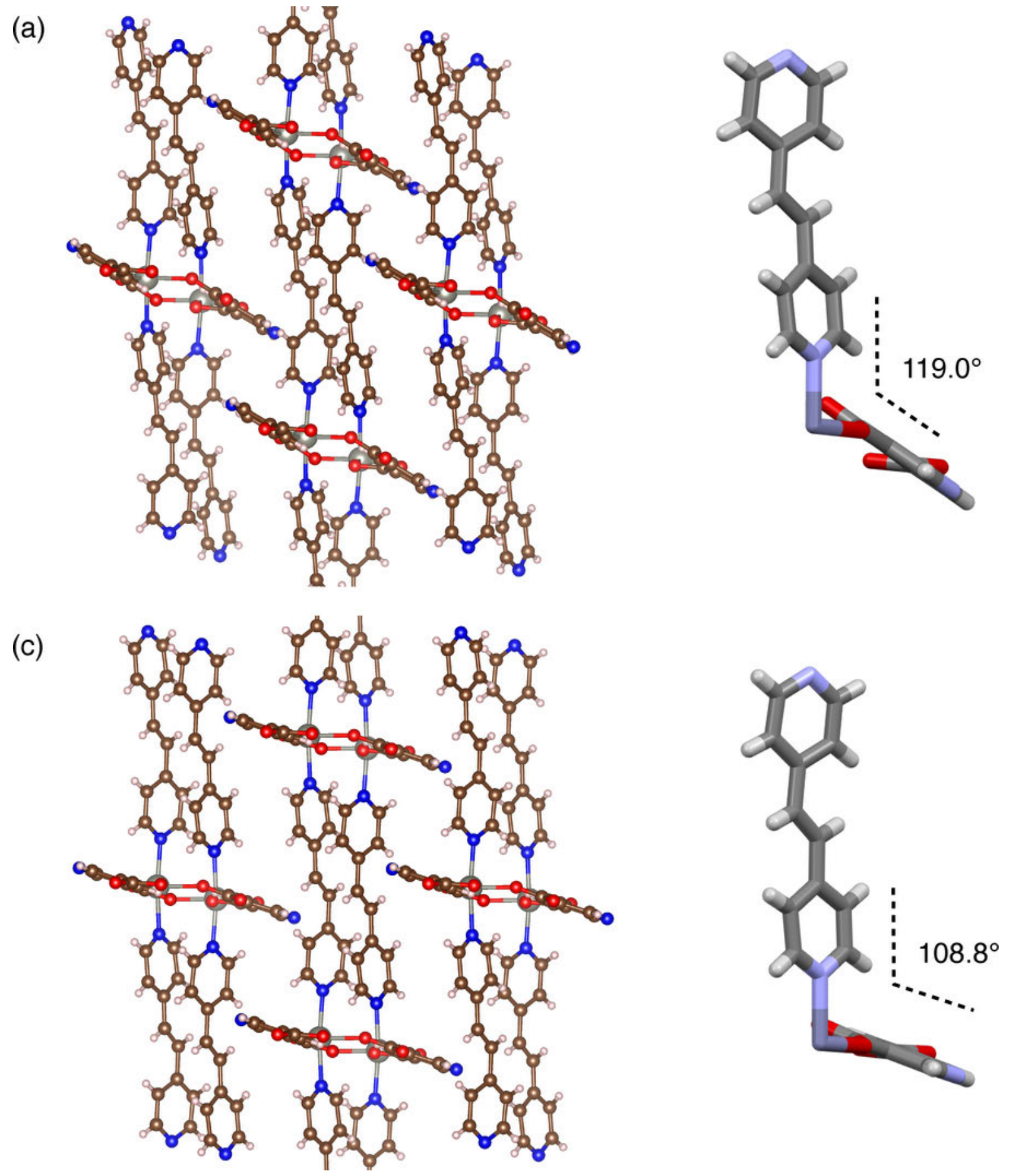

Figure S4. Crystal structures of degassed **2** determined by SXRD at (a) 123 K and (c) 298 K, showing both the packing structure (left) and the asymmetric unit (right). The unit cell volume increased from 897.3 Å^3 at 123 K to 964.7 Å^3 at 298 K, reflecting thermal expansion of the framework (see Table S1). The angle between the pillar and layer ligands decreased from 119.0° at 123 K to 108.8° at 298 K.

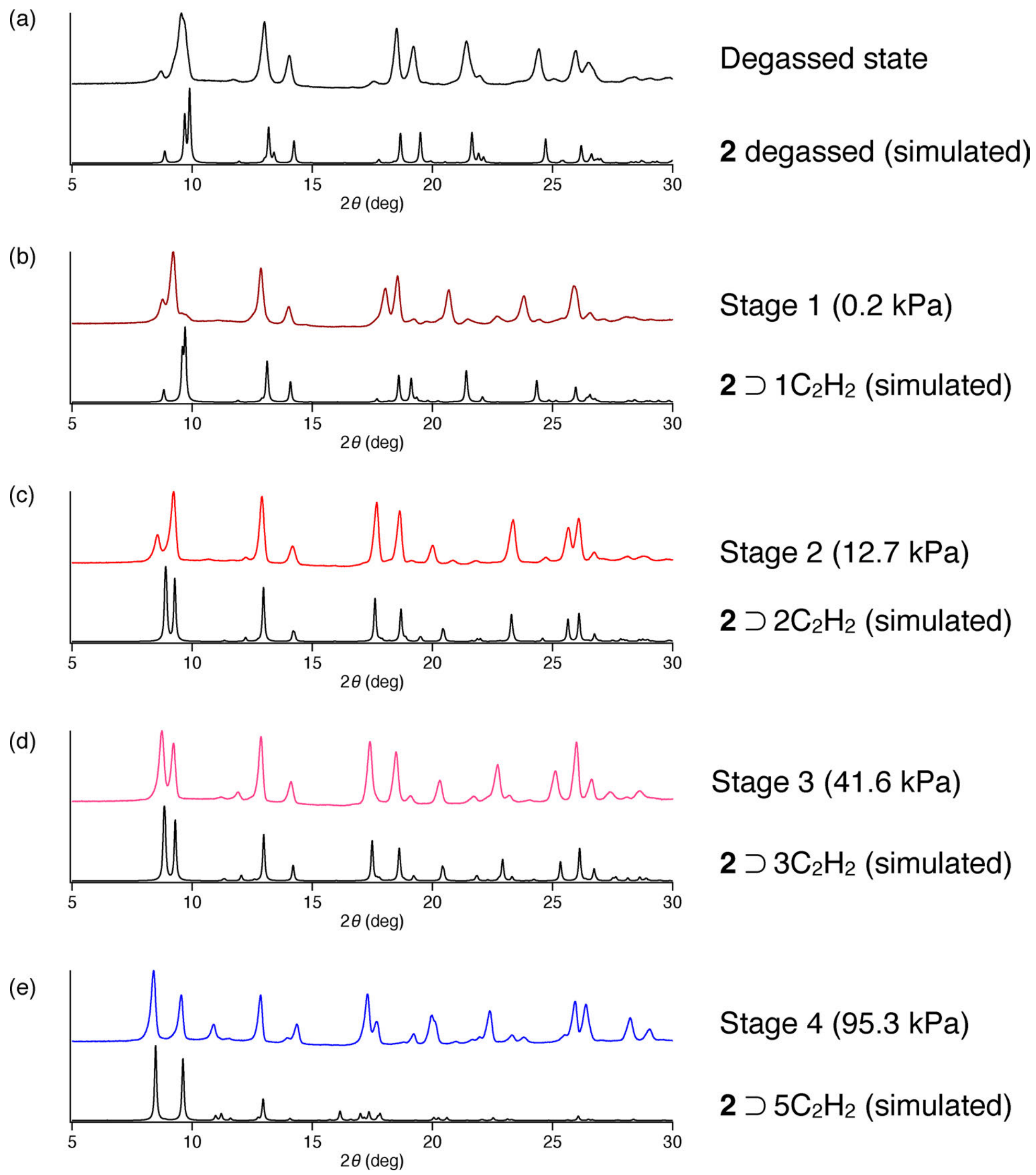

Figure S5. PXRD data of **2** at different acetylene adsorption steps. PXRD patterns of **2** corresponding to (a) the degassed state, (b) Stage 1 (0.2 kPa), (c) Stage 2 (12.7 kPa), (d) Stage 3 (41.6 kPa), and (e) Stage 4 (95.3 kPa), measured at 195 K. Each experimental PXRD pattern is shown together with the simulated pattern based on the corresponding crystal structure: (a) degassed **2**, (b) **2**$\supset$1C_2H_2, (c) **2**$\supset$2C_2H_2, (d) **2**$\supset$3C_2H_2, and (e) **2**$\supset$5C_2H_2 per Zn_2. Simulated PXRD patterns for Stages 1–4 were generated from the crystal structure data determined at 195 K (Table S1). The simulated pattern for the degassed phase in panel (a) was generated from the SXRD structure determined at 123 K (Table S1).

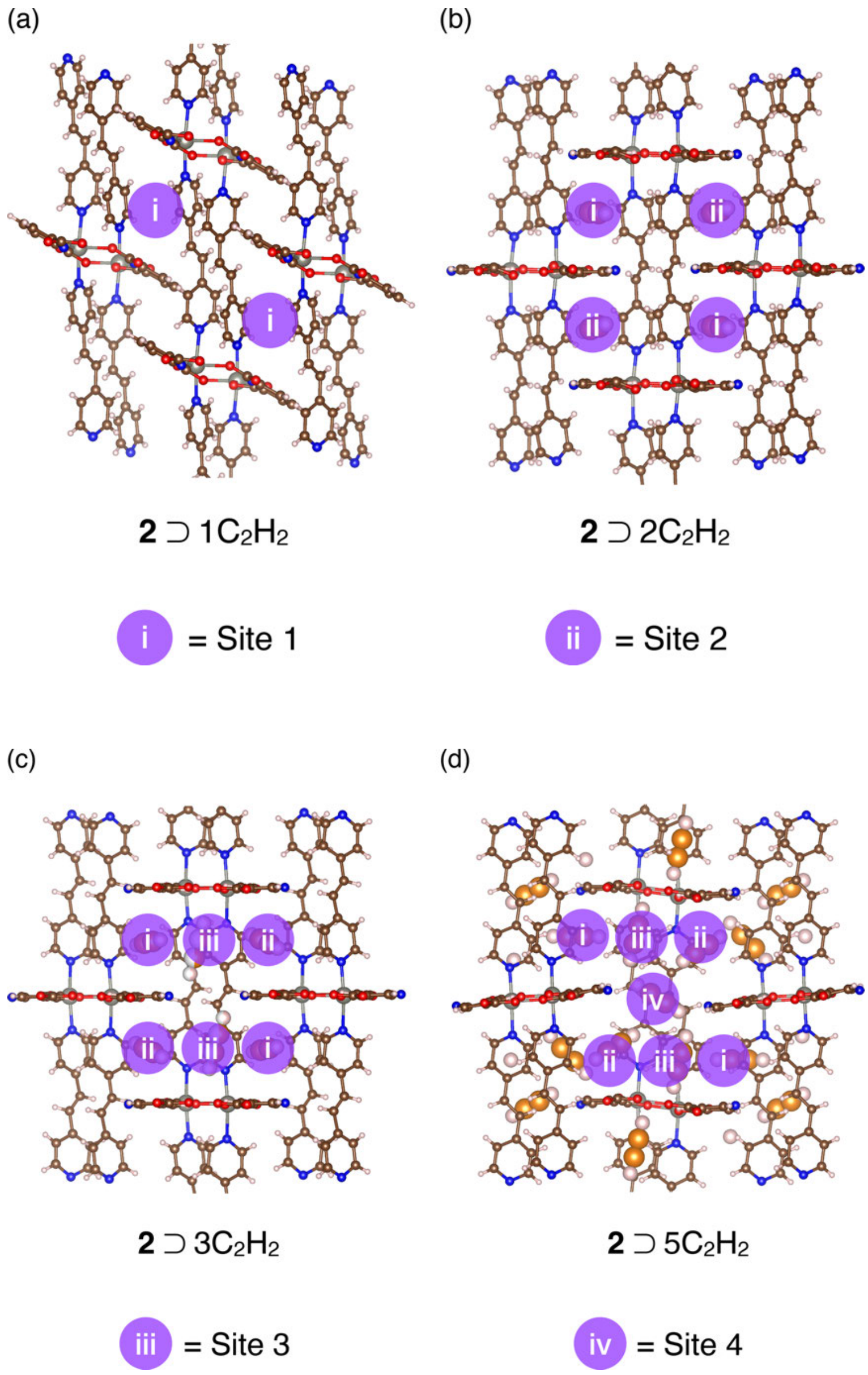

Figure S6. Schematic illustration of the acetylene adsorption sites (i–iv) in **2**, generated at (a) Stage 1 (1 C_2H_2 per Zn_2), (b) Stage 2 (2 C_2H_2 per Zn_2), (c) Stage 3 (3 C_2H_2 per Zn_2), and (d) Stage 4 (5 C_2H_2 per Zn_2).

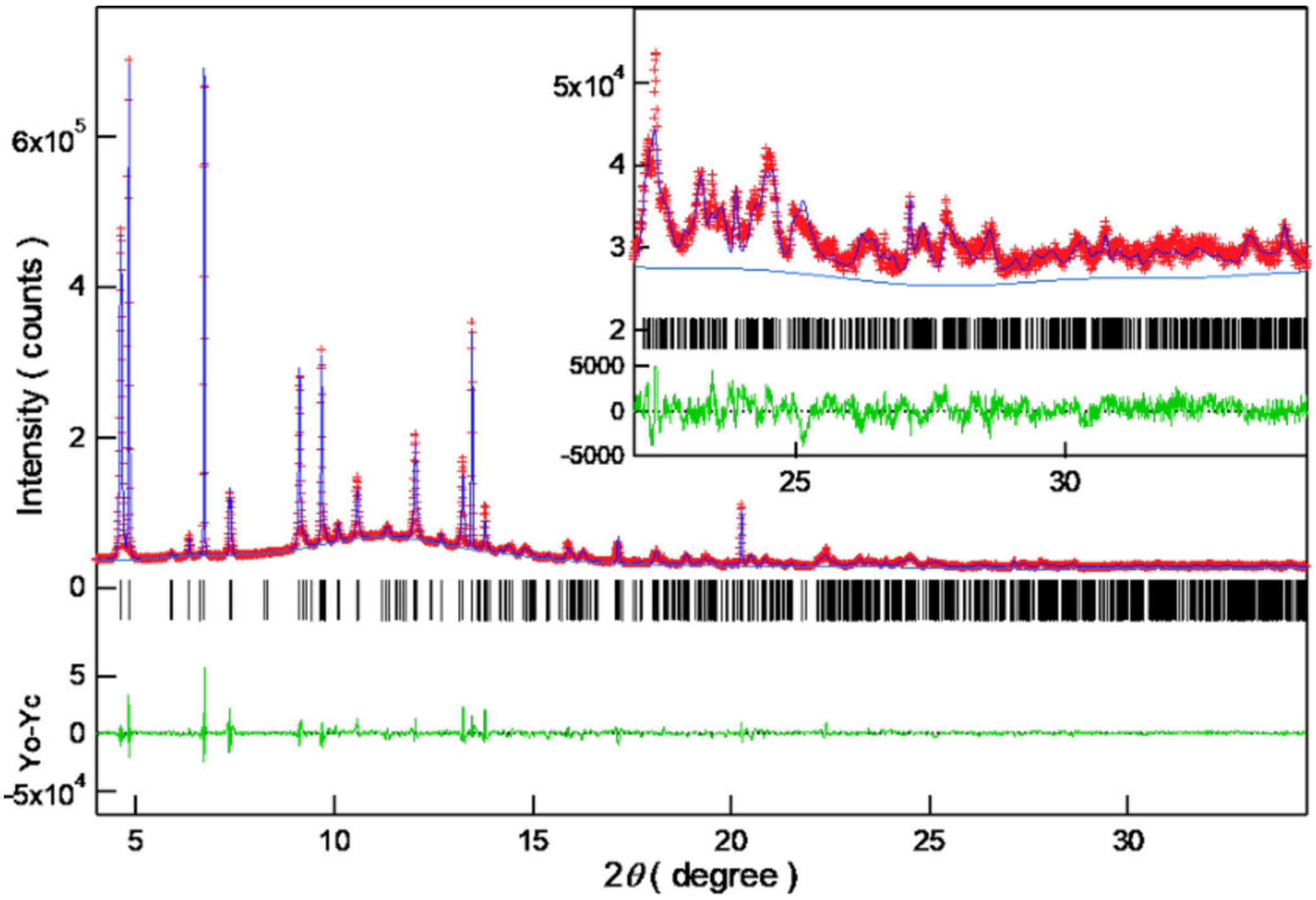

Figure S7. Fitting result of the Rietveld refinement for **2** at Stage 2 (2 C_2H_2 per Zn_2), measured at 195 K.

(a)

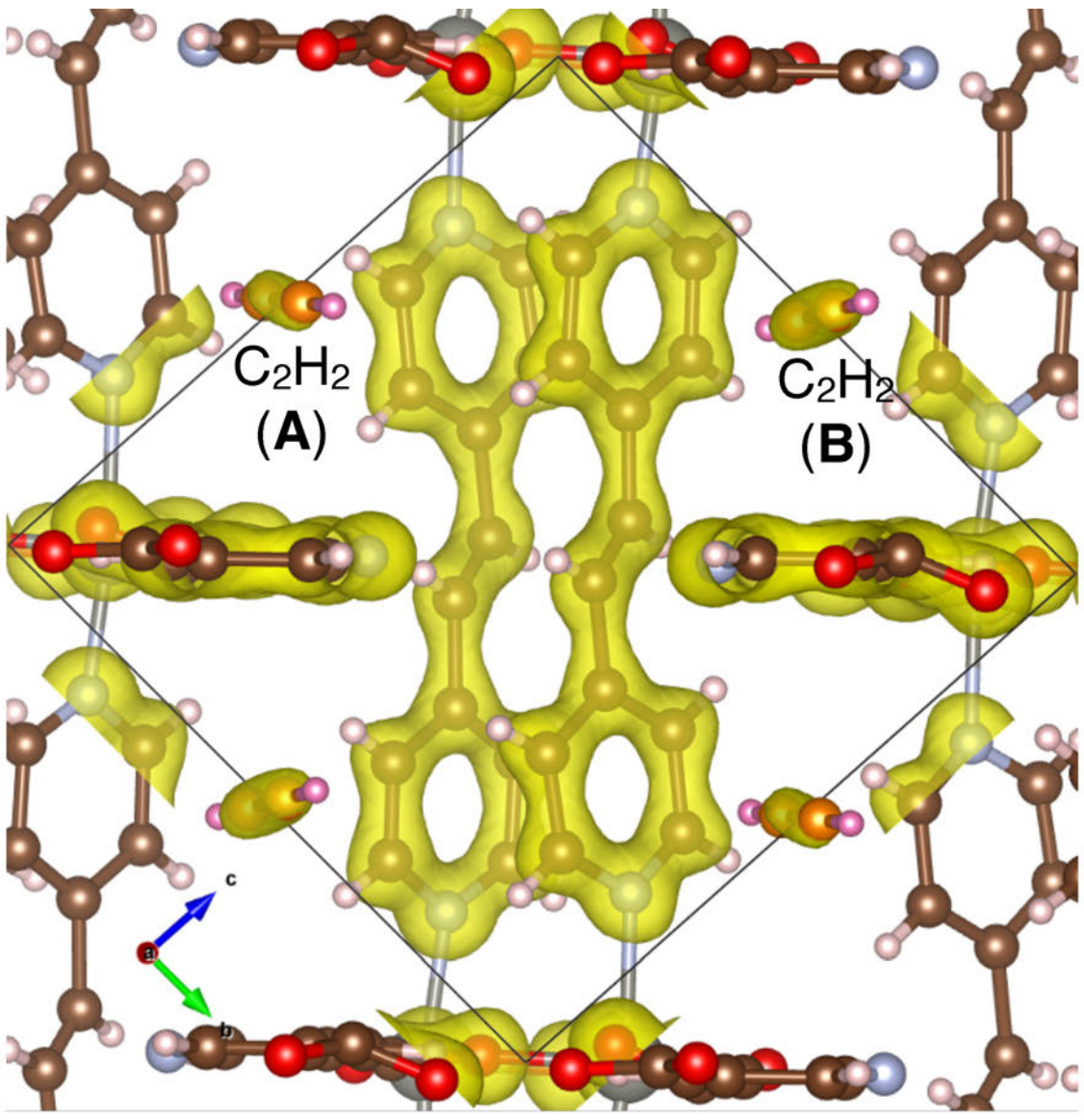

(b)

	B (Å²)
Zn	1.18(7)
Framework	3.1(2)
C_2H_2 (**A**)	23(1)
C_2H_2 (**B**)	23(1)

(c)

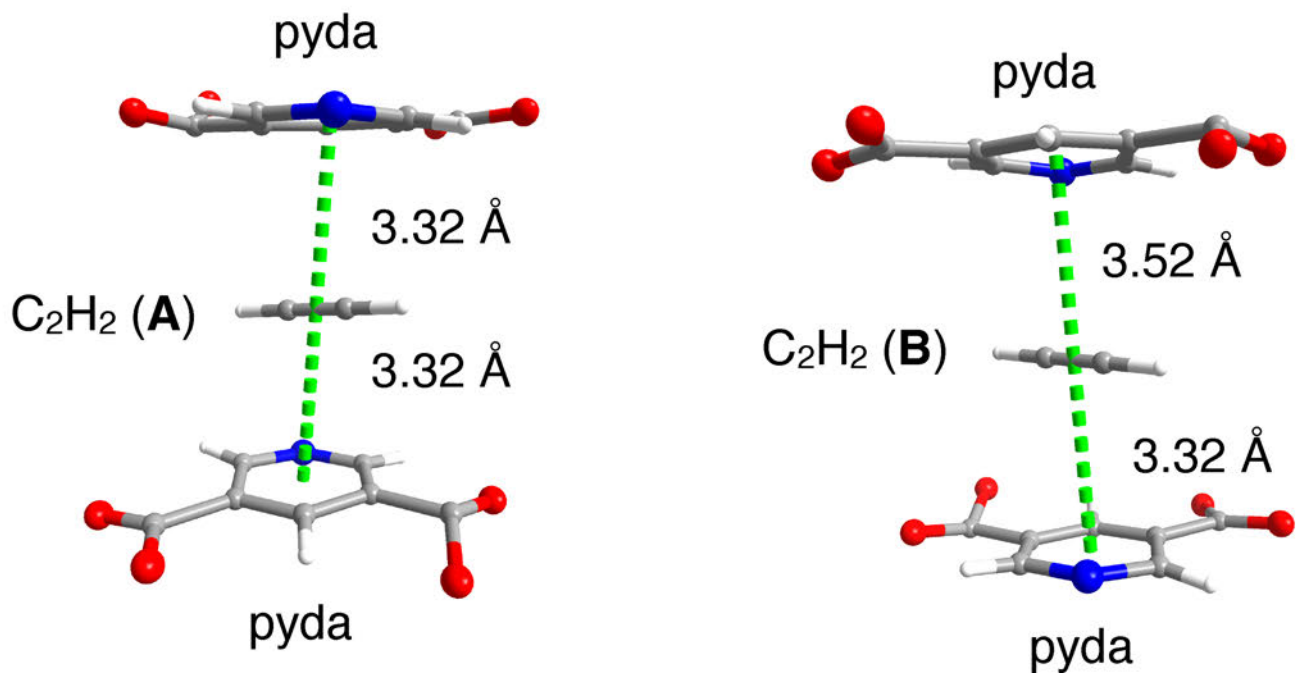

Figure S8. (a) MEM charge density map of **2** at Stage 2 (2 C_2H_2 per Zn_2) at 195 K (R_{MEM} = 2.82%; isosurface level 1.6 e Å^{-3}). (b) Isotropic temperature factor *B* of Zn atom, framework, and adsorbed C_2H_2 molecules. (c) Intermolecular distances between the adsorbed acetylene molecules and framework ligands in **2** at Stage 2 (195 K). The distances between the adsorbed acetylene molecules and the aromatic rings of the pyda ligands are approximately 3.3–3.5 Å, suggesting the presence of host–guest π interactions that contribute to stabilization of the adsorbed state.

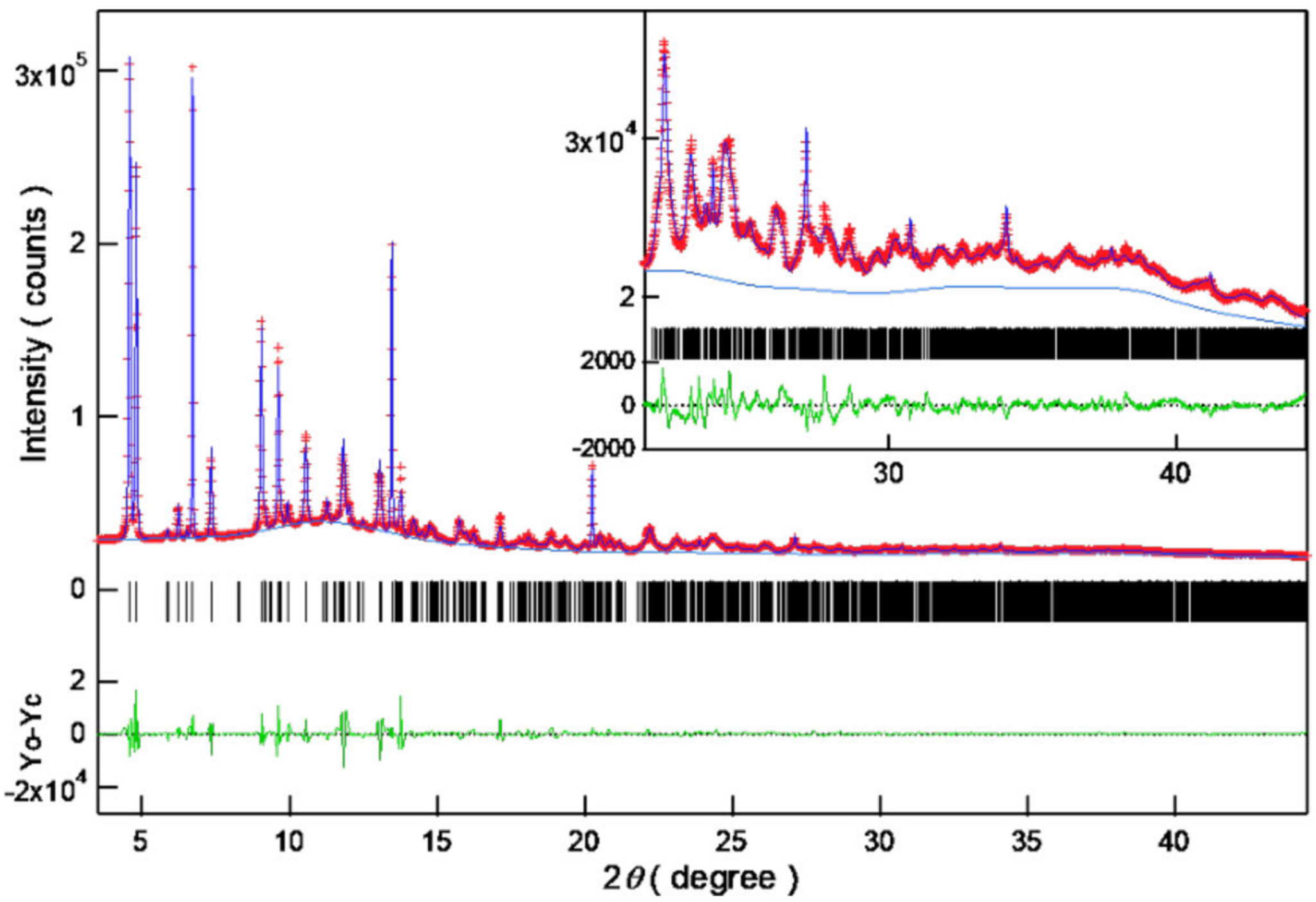

Figure S9. Fitting result of the Rietveld refinement for **2** at Stage 3 (3 C_2H_2 per Zn_2), measured at 195 K.

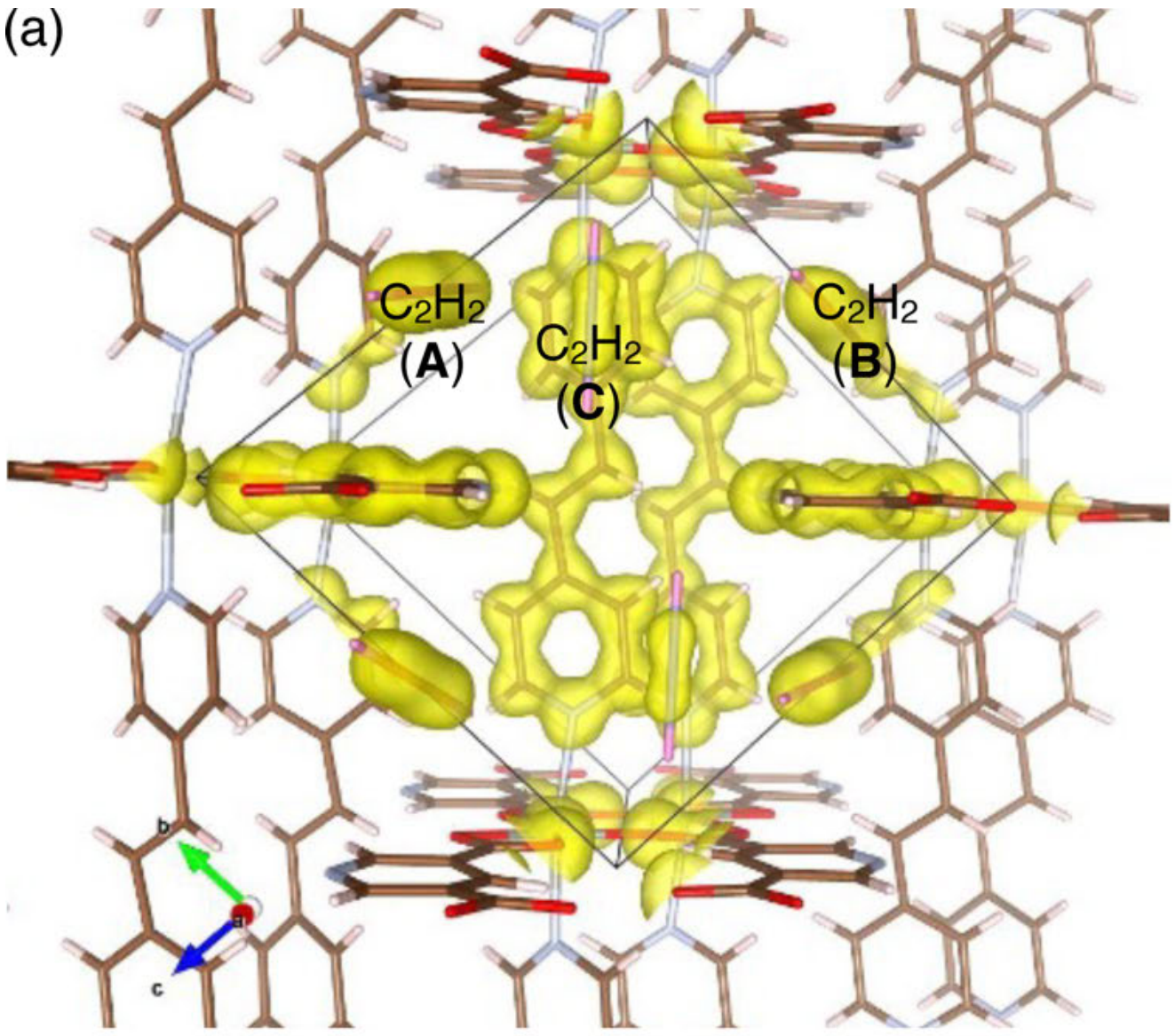

(b)

	B (Å^2)
Zn	0.86(8)
Framework	1.9(2)
C_2H_2 (**A**)	10(1)
C_2H_2 (**B**)	10(1)
C_2H_2 (**C**)	10(1)

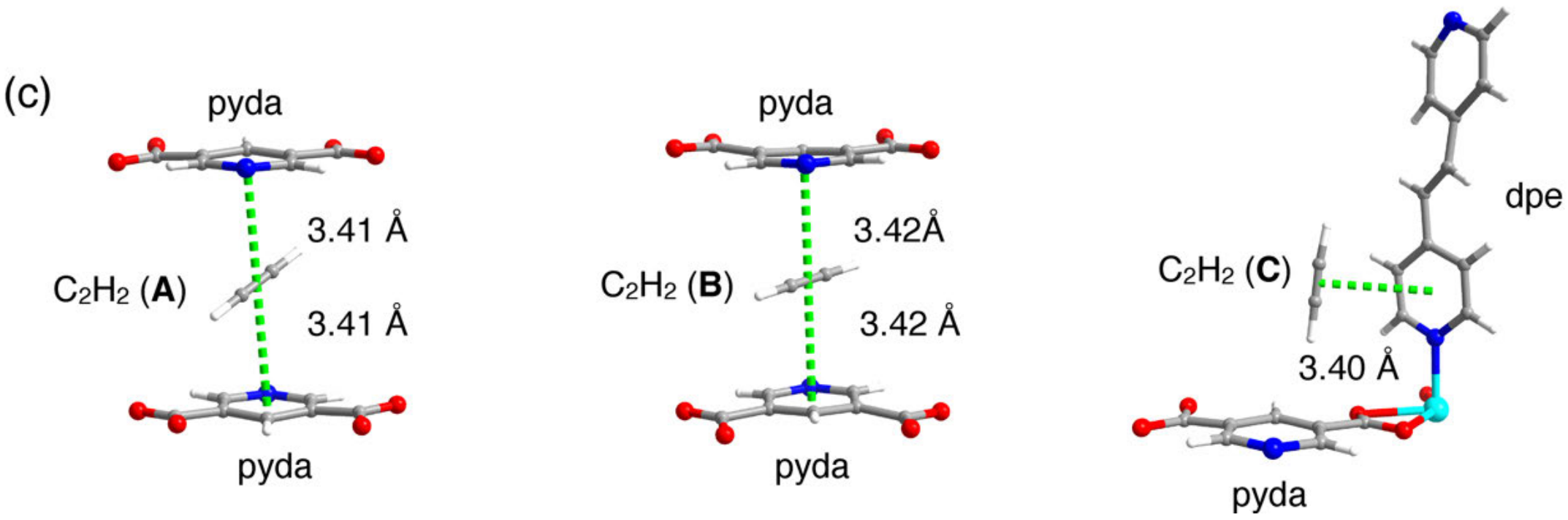

Figure S10. (a) MEM charge density map of **2** at Stage 3 (3 C_2H_2 per Zn_2) at 195 K (R_{MEM} = 2.74%; isosurface level 1.5 e Å^{-3}). (b) Isotropic temperature factor B of Zn atoms, framework, and adsorbed C_2H_2 molecules. (c) Intermolecular distances between the adsorbed acetylene molecules and framework ligands in **2** at Stage 3 (195 K). Two acetylene molecules (**A** and **B**) are stabilized through π interactions with the pyda ligands of the framework, while a third acetylene molecule (**C**) occupies a site between them and is likely stabilized by π interactions with the dpe ligand.

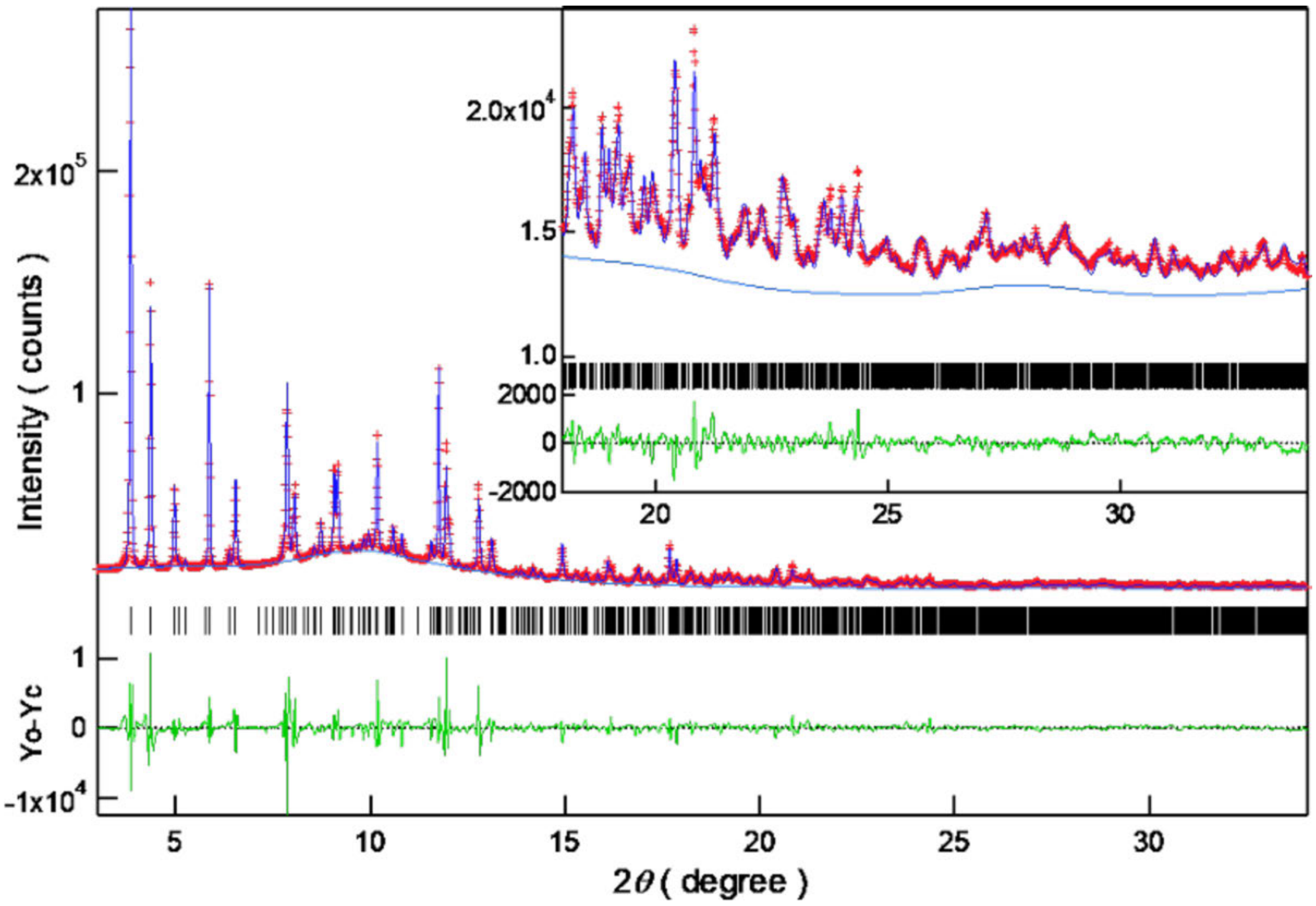

Figure S11. Fitting result of the Rietveld refinement for **2** at Stage 4 (5 C_2H_2 per Zn_2), measured at 195 K.

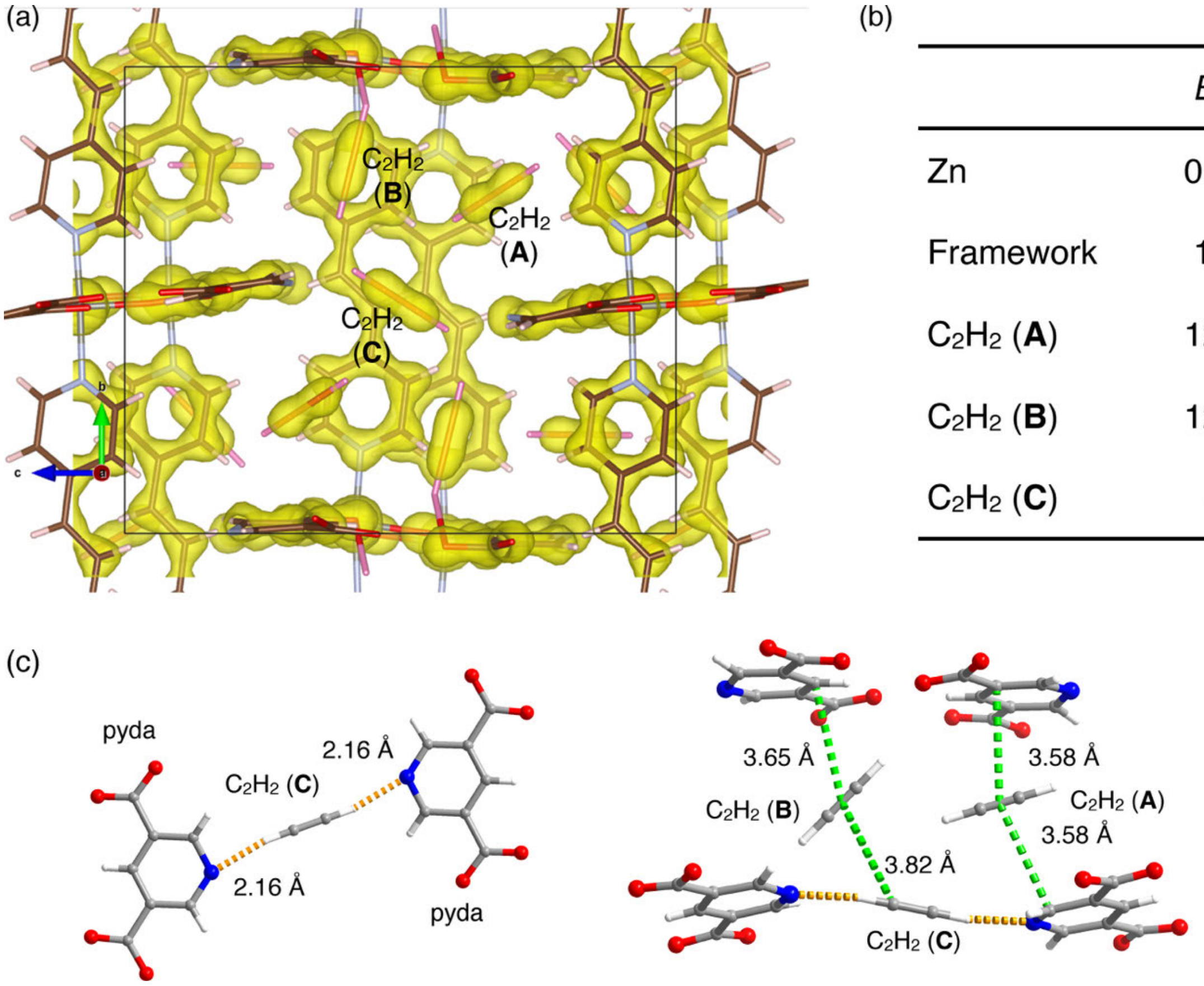

(b)

	B (Å^2)
Zn	0.84(8)
Framework	1.1(2)
C_2H_2 (**A**)	12.7(9)
C_2H_2 (**B**)	12.7(9)
C_2H_2 (**C**)	3(1)

Figure S12. (a) MEM charge density map of **2** at Stage 4 (5 C_2H_2 per Zn_2) at 195 K (R_{MEM} = 4.51%; isosurface level 1.5 e Å^{-3}). (b) Isotropic temperature factor *B* of Zn atoms, framework, and adsorbed C_2H_2 molecules. (c) Intermolecular distances between the adsorbed acetylene molecules and framework ligands in **2** at Stage 4 (195 K). One acetylene molecule is inserted between two neighboring pyda ligands and is stabilized through hydrogen bonding with the pyridyl nitrogen atoms. This interaction stabilizes the highly expanded framework, creating additional pore volume that accommodates extra acetylene molecules through weaker van der Waals interactions.

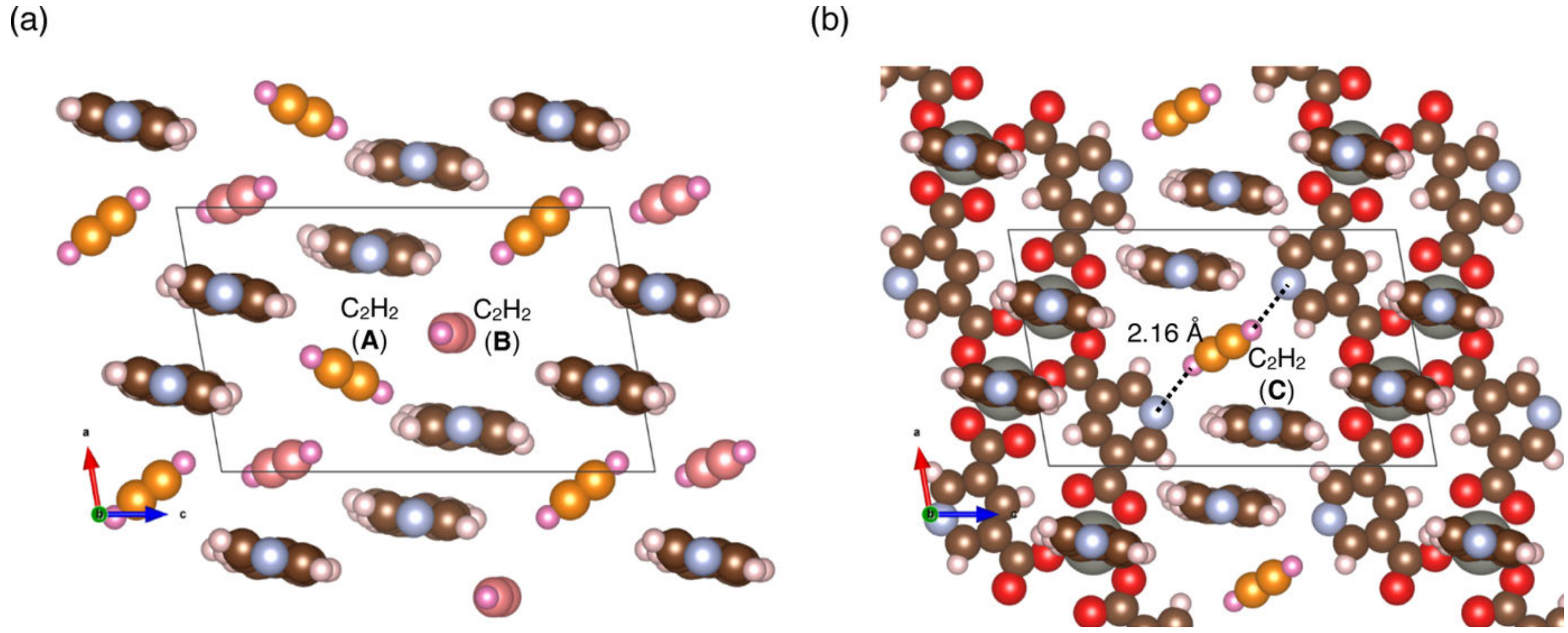

Figure S13. Crystal structures of **2** at Stage 4 (5 C_2H_2 per Zn_2) at 195 K, highlighting (a) acetylene molecules adsorbed at Site 1 and 2 (C_2H_2 **A**), and 3 (C_2H_2 **B**), and (b) an acetylene molecule adsorbed in Site 4 (C_2H_2 **C**). The acetylene at Site 4 (C_2H_2 **C**) forms hydrogen bonds (2.163 Å) with nitrogen atoms between opposing pyda ligands.

2. Supporting Table

Table S1. SXRD crystallographic data and structural refinement summary.

	**1**⊃DMF	**2**⊃DMF	**2** (degassed) 123K	**2** (degassed) 298K	**2**⊃1C_2H_2 (Stage 1)
Crystal System	Monoclinic	Monoclinic	Triclinic	Triclinic	Triclinic
Space Group	$P2_1/c$	$C2/m$	P-1	P-1	P-1
a (Å)	10.0822(15)	15.444(2)	9.7116(13)	10.0924(12)	9.850(4)
b (Å)	13.6618(19)	13.663(2)	10.0677(10)	10.1008(15)	10.111(4)
c (Å)	15.261(2)	10.1149(19)	10.1437(8)	10.1904(11)	10.186(4)
α (°)	90	90	79.582(7)	84.612(10)	79.684(6)
β (°)	105.516(3)	108.463(6)	86.814(9)	78.327(9)	86.215(7)
γ (°)	90	90	66.933(11)	71.570(12)	67.558(5)
V (Å^3)	2025.5(5)	2024.5(6)	897.32(18)	964.7(2)	922.5(6)
Z	4	2	2	2	2
Diffractometer	Rigaku XtaLAB P200	Rigaku XtaLAB P200	Rigaku XtaLAB P200	Rigaku XtaLAB P200	Synchrotron X-ray (SPring-8)
μ (cm^{-1})	1.238	1.240	1.399	1.301	0.321
Radiation type	Mo Kα	Mo Kα	Mo Kα	Mo Kα	Synchrotron
Radiation wavelength (Å)	0.71075	0.71075	0.71075	0.71075	0.4139
F(000)	840	868	420	420	420
Goodness of fit	1.150	1.099	1.003	0.933	1.157
Temperature (K)	123	93	123	298	195
Reflections collected	17307	16002	16805	17805	4486
Independent reflections	4493	2424	4975	5351	2697
R_1 (I>2.00σ(I))	0.1333	0.0514	0.0704	0.0871	0.1057
wR_2	0.3059	0.1464	0.1774	0.2352	0.3186
Void space (Å^3)[a]	383.9	364.1 [c]	65.06	88.60	77.77
Void space per unit cell[b]	19.0%	18.0% [c]	7.3%	9.2%	8.4%
CCDC number	2496652	2496653	2496651	2496650	2496649

[a]The void space was calculated using Mercury software based on the structure model with all guest molecules removed (contact surface; probe radius = 1.2 Å).
[b]Void fraction.
[c]Void space was calculated using the representative ordered model. The disordered-included crystallographic model gives a lower value (15.3%) because the disordered components partially occupy the pore space.

Table S2. Summary of crystal structure data obtained from SR-PXRD analysis.

	**2**⊃2C_2H_2 (Stage 2)	**2**⊃3C_2H_2 (Stage 3)	**2**⊃5C_2H_2 (Stage 4)
Crystal System	Triclinic	Triclinic	Monoclinic
Space Group	*P*-1	*P*-1	$P2_1/n1$
a (Å)	10.0667(2)	10.0598(3)	10.0935(2)
b (Å)	10.2292(5)	10.3000(7)	13.6605(2)
c (Å)	10.2663(5)	10.3711(7)	16.3608(5)
α (°)	83.480(2)	82.522(2)	90
β (°)	76.215(4)	75.643(9)	99.788(2)
γ (°)	75.890(4)	75.935(9)	90
V (Å^3)	994.02(8)	1007.0 (1)	2223.0(1)
Z	2	2	4
Radiation type	Synchrotron	Synchrotron	Synchrotron
Radiation wavelength (Å)	0.799599(1)	0.799384(1)	0.699692(1)
Temperature (K)	195	195	195
R_{wp}	0.0386	0.0305	0.0278
R_I	0.0506	0.0439	0.0416
Void space (Å^3) [a]	214.22	274.08	703.15
Void space per unit cell[b]	21.6%	27.2%	31.6%
CCDC number	2558775	2558972	2558977

[a]The void space was calculated using Mercury software based on the structure model with all guest molecules removed (contact surface; probe radius = 1.2 Å).
[b]Void fraction.

Table S3. Structure parameters of **2**⊃2C_2H_2.[a]

Site	Occupancy	*x*	*y*	*z*	*B* [Å^2]
**Zn**	1	0.6102(6)	1.0933(8)	0.0953(8)	1.18(7)
**O1**	1	-0.154(2)	1.060(2)	0.106(2)	3.1(2)
**O2**	1	0.416(2)	1.150(3)	0.166(3)	3.1(2)
**O3**	1	-0.312(2)	1.222(3)	0.223(3)	3.1(2)
**O4**	1	0.330(2)	1.048(3)	0.033(4)	3.1(2)
**C1**	1	-0.197(3)	1.175(3)	0.149(3)	3.1(2)
**C19**	1	-0.082(4)	1.216(6)	0.193(5)	3.1(2)
**C4**	1	-0.115(3)	1.305(5)	0.294(5)	3.1(2)
**H3**	1	-0.210(3)	1.337(5)	0.339(5)	3.1(2)
**N2**	1	-0.008(3)	1.337(4)	0.333(4)	3.1(2)
**C3**	1	0.130(3)	1.290(5)	0.274(5)	3.1(2)
**H2**	1	0.200(3)	1.328(5)	0.294(5)	3.1(2)
**C16**	1	0.167(3)	1.193(6)	0.181(6)	3.1(2)
**C17**	1	0.317(2)	1.127(7)	0.123(6)	3.1(2)
**C18**	1	0.056(4)	1.161(5)	0.141(5)	3.1(2)
**H13**	1	0.076(4)	1.084(5)	0.090(5)	3.1(2)
**C2**	1	0.677(3)	0.566(2)	0.528(2)	3.1(2)
**H1**	1	0.769(3)	0.510(2)	0.515(2)	3.1(2)
**C15**	1	0.572(3)	0.541(2)	0.627(2)	3.1(2)
**H12**	1	0.480(3)	0.595(2)	0.637(2)	3.1(2)
**N1**	1	0.608	0.921	0.266	3.1(2)
**C9**	1	0.718	0.818	0.237	3.1(2)
**H6**	1	0.783	0.825	0.154	3.1(2)
**C13**	1	0.741	0.703	0.317	3.1(2)
**H10**	1	0.824	0.635	0.292	3.1(2)
**C5**	1	0.642	0.688	0.436	3.1(2)
**C12**	1	0.526	0.791	0.464	3.1(2)
**H9**	1	0.457	0.785	0.544	3.1(2)
**C7**	1	0.510	0.905	0.376	3.1(2)
**H4**	1	0.426	0.973	0.395	3.1(2)
**C6**	1	0.597	0.427	0.731	3.1(2)
**C14**	1	0.716	0.324	0.716	3.1(2)
**H11**	1	0.790	0.322	0.639	3.1(2)
**C8**	1	0.723	0.220	0.819	3.1(2)
**H5**	1	0.802	0.147	0.808	3.1(2)
**N3**	1	0.624	0.222	0.929	3.1(2)
**C10**	1	0.509	0.322	0.939	3.1(2)
**H7**	1	0.436	0.322	1.017	3.1(2)
**C11**	1	0.491	0.421	0.846	3.1(2)

**H8**	1	0.405	0.488	0.856	3.1(2)
**C1 (A)**	1	0.044	0.511	0.023	23(1)
**H1 (A)**	1	0.122	0.531	0.067	23(1)
**C1 (B)**	1	0.046	0.018	0.515	23(1)
**H1 (B)**	1	0.128	0.050	0.540	23(1)

[a](A) and (B) denote the atoms of the acetylene molecule adsorbed at the corresponding sites.

Table S4. Structure parameters of **2**⊃$3C_2H_2$.[a]

Site	Occupancy	*x*	*y*	*z*	*B* [Å^2]
**Zn1**	1	0.6059(7)	1.0893(9)	0.088(1)	0.86(8)
**O2**	1	0.400(1)	1.176(3)	0.163(3)	1.9(2)
**O4**	1	0.346(2)	1.043(4)	0.046(4)	1.9(2)
**C7**	1	0.310(1)	1.133(6)	0.125(6)	1.9(2)
**C10**	1	-0.198(2)	1.167(7)	0.153(6)	1.9(2)
**O1**	1	-0.158(2)	1.088(3)	0.062(3)	1.9(2)
**O3**	1	-0.321(2)	1.223(4)	0.207(4)	1.9(2)
**C28**	1	0.592(4)	0.591(2)	0.632(3)	1.9(2)
**H28**	1	0.529(4)	0.672(2)	0.660(3)	1.9(2)
**C11**	1	0.670(3)	0.580(2)	0.509(2)	1.9(2)
**H11**	1	0.743(3)	0.504(2)	0.485(2)	1.9(2)
**C6**	1	0.158(1)	1.187(6)	0.189(5)	1.9(2)
**C13**	1	0.123(1)	1.279(6)	0.285(6)	1.9(2)
**H13**	1	0.195(1)	1.304(6)	0.314(6)	1.9(2)
**C8**	1	0.053(1)	1.145(5)	0.153(5)	1.9(2)
**H8**	1	0.077(1)	1.072(5)	0.097(5)	1.9(2)
**N18**	1	-0.015(2)	1.337(5)	0.335(5)	1.9(2)
**C14**	1	-0.120(1)	1.301(5)	0.292(5)	1.9(2)
**H14**	1	-0.216(1)	1.343(5)	0.324(5)	1.9(2)
**C9**	1	-0.085(1)	1.206(6)	0.199(6)	1.9(2)
**N5**	1	0.598(4)	0.245(2)	0.897(2)	1.9(2)
**C20**	1	0.668(4)	0.233(2)	0.769(2)	1.9(2)
**H20**	1	0.725(4)	0.149(2)	0.741(2)	1.9(2)
**C23**	1	0.501(4)	0.358(3)	0.932(2)	1.9(2)
**H23**	1	0.438(4)	0.358(3)	1.017(2)	1.9(2)
**C27**	1	0.664(4)	0.345(2)	0.679(2)	1.9(2)
**H27**	1	0.717(4)	0.338(2)	0.590(2)	1.9(2)
**C24**	1	0.494(3)	0.471(2)	0.845(2)	1.9(2)
**H24**	1	0.431(3)	0.553(2)	0.874(2)	1.9(2)
**C16**	1	0.579(4)	0.465(2)	0.719(2)	1.9(2)
**C15**	1	0.652(3)	0.702(3)	0.414(3)	1.9(2)
**C25**	1	0.532(2)	0.803(3)	0.440(3)	1.9(2)
**H25**	1	0.461(2)	0.795(3)	0.518(3)	1.9(2)
**C26**	1	0.750(2)	0.711(2)	0.296(3)	1.9(2)
**H26**	1	0.833(2)	0.642(2)	0.278(3)	1.9(2)
**C19**	1	0.518(3)	0.916(3)	0.352(3)	1.9(2)
**H19**	1	0.438(3)	0.988(3)	0.373(3)	1.9(2)
**C21**	1	0.730(3)	0.821(3)	0.206(2)	1.9(2)
**H21**	1	0.793(4)	0.823(3)	0.121(2)	1.9(2)

**N12**	1	0.615(4)	0.926(3)	0.239(3)	1.9(2)
**H1 (A)**	1	0.093	0.609	0.009	10(1)
**C1 (A)**	1	0.033	0.539	0.003	10(1)
**H1 (A)**	1	0.114	0.036	0.572	10(1)
**C1 (B)**	1	0.041	0.013	0.526	10(1)
**H1 (B)**	0.5	-0.045	0.669	0.451	10(1)
**C1 (C)**	0.5	-0.034	0.745	0.373	10(1)
**C2 (C)**	0.5	-0.020	0.832	0.285	10(1)
**H2 (C)**	0.5	-0.008	0.909	0.207	10(1)

[a](A), (B), and (C) denote the atoms of the acetylene molecule adsorbed at the corresponding sites.

Table S5. Structure parameters of **2**⊃5C_2H_2.

Site	Occupancy	*x*	*y*	*z*	*B* [Å^2]
**Zn**	1	0.1763(5)	0.0195(5)	0.5821(3)	0.84(8)
**O1**	1	0.602(2)	-0.024(2)	0.430(1)	1.1(2)
**O2**	1	-0.025(2)	-0.017(2)	0.344(1)	1.1(2)
**O3**	1	0.667(1)	-0.020(2)	0.302(1)	1.1(2)
**O4**	1	0.131(2)	0.006(2)	0.458(1)	1.1(2)
**C1**	1	0.576(2)	-0.019(3)	0.352(1)	1.1(2)
**C17**	1	0.096(2)	-0.007(4)	0.381(1)	1.1(2)
**C19**	1	0.431(1)	-0.022(3)	0.313(1)	1.1(2)
**C4**	1	0.393(2)	-0.029(3)	0.228(1)	1.1(2)
**H3**	1	0.454(2)	-0.041(3)	0.192(1)	1.1(2)
**N2**	1	0.262(2)	-0.024(2)	0.1943(8)	1.1(2)
**C3**	1	0.166(1)	-0.026(3)	0.243(1)	1.1(2)
**H2**	1	0.078(1)	-0.046(3)	0.218(1)	1.1(2)
**C16**	1	0.201(2)	-0.015(4)	0.327(1)	1.1(2)
**C18**	1	0.334(2)	-0.013(4)	0.3630(8)	1.1(2)
**H13**	1	0.359(2)	-0.003(4)	0.4211(8)	1.1(2)
**N1**	1	0.168(4)	0.1774(9)	0.571(2)	1.1(2)
**C9**	1	0.192(4)	0.219(1)	0.500(1)	1.1(2)
**H6**	1	0.215(4)	0.178(1)	0.458(1)	1.1(2)
**C13**	1	0.180(3)	0.320(1)	0.490(1)	1.1(2)
**H10**	1	0.174(3)	0.349(1)	0.437(1)	1.1(2)
**C5**	1	0.160(4)	0.377(1)	0.556(1)	1.1(2)
**C12**	1	0.148(4)	0.333(1)	0.630(1)	1.1(2)
**H9**	1	0.144(4)	0.374(1)	0.677(1)	1.1(2)
**C7**	1	0.152(4)	0.233(1)	0.637(1)	1.1(2)
**H4**	1	0.123(4)	0.202(1)	0.682(1)	1.1(2)
**C2**	1	0.156(3)	0.488(1)	0.548(1)	1.1(2)
**H1**	1	0.152(3)	0.514(1)	0.493(1)	1.1(2)
**C15**	1	0.178(3)	0.556(1)	0.609(1)	1.1(2)
**H12**	1	0.180(3)	0.531(1)	0.663(1)	1.1(2)
**C6**	1	0.177(3)	0.663(1)	0.600(1)	1.1(2)
**C14**	1	0.196(4)	0.705(1)	0.527(1)	1.1(2)
**H11**	1	0.199(4)	0.664(1)	0.480(1)	1.1(2)
**C8**	1	0.190(4)	0.805(1)	0.518(1)	1.1(2)
**H5**	1	0.204(4)	0.834(1)	0.467(1)	1.1(2)
**N3**	1	0.176(4)	0.862(1)	0.583(2)	1.1(2)
**C10**	1	0.145(4)	0.822(1)	0.653(1)	1.1(2)
**H7**	1	0.131(4)	0.859(1)	0.700(1)	1.1(2)
**C11**	1	0.148(4)	0.721(1)	0.663(1)	1.1(2)

**H8**	1	0.122(4)	0.697(1)	0.712(1)	1.1(2)
**aH1**	1	0.030	0.191	0.073	12.7(9)
**aC1**	1	0.069	0.208	0.135	12.7(9)
**aC2**	1	0.113	0.227	0.206	12.7(9)
**aH2**	1	0.152	0.244	0.269	12.7(9)
**bH1**	1	0.450	0.310	0.296	12.7(9)
**bC1**	1	0.465	0.262	0.347	12.7(9)
**bC2**	1	0.482	0.206	0.404	12.7(9)
**bH2**	1	0.497	0.157	0.455	12.7(9)
**cH1**	1	0.400	0.474	0.410	3(1)
**cC1**	1	0.464	0.490	0.467	3(1)

[a](A), (B), and (C) denote the atoms of the acetylene molecule adsorbed at the corresponding sites.
